# Copper‐Zinc Bimetallic Single‐Atom Catalysts with Localized Surface Plasmon Resonance‐Enhanced Photothermal Effect and Catalytic Activity for Melanoma Treatment and Wound‐Healing

**DOI:** 10.1002/advs.202207342

**Published:** 2023-04-25

**Authors:** Lidan Liu, Haifeng Zhang, Shun Xing, Yu Zhang, Li Shangguan, Chao Wei, Feng Peng, Xuanyong Liu

**Affiliations:** ^1^ State Key Laboratory of High Performance Ceramics and Superfine Microstructure Shanghai Institute of Ceramics Chinese Academy of Sciences Shanghai 200050 China; ^2^ Center of Materials Science and Optoelectronics Engineering University of Chinese Academy of Sciences Beijing 100049 China; ^3^ School of Chemistry and Materials Science Hangzhou Institute for Advanced Study University of Chinese Academy of Sciences 1 Sub‐lane Xiangshan Hangzhou 310024 China; ^4^ Medical Research Institute Department of Orthopedics Guangdong Provincial People's Hospital (Guangdong Academy of Medical Sciences) Southern Medical University Guangzhou 510080 China

**Keywords:** bimetallic single‐atom, localized surface plasmon resonance, melanoma therapy, photothermal combined chemodynamic therapy, wound healing

## Abstract

Nanomaterials with photothermal combined chemodynamic therapy (PTT‐CDT) have attracted the attention of researchers owing to their excellent synergistic therapeutic effects on tumors. Thus, the preparation of multifunctional materials with higher photothermal conversion efficiency and catalytic activity can achieve better synergistic therapeutic effects for melanoma. In this study, a Cu–Zn bimetallic single‐atom (Cu/PMCS) is constructed with augmented photothermal effect and catalytic activity due to the localized surface plasmon resonance (LSPR) effect. Density functional theory calculations confirmed that the enhanced photothermal effect of Cu/PMCS is due to the appearance of a new d‐orbital transition with strong spin‐orbit coupling and the induced LSPR. Additionally, Cu/PMCS exhibited increased catalytic activity in the Fenton‐like reaction and glutathione depletion capacity, further enhanced by increased temperature and LSPR. Consequently, Cu/PMCS induced better synergistic anti‐melanoma effects via PTT‐CDT than PMCS in vitro and in vivo. Furthermore, compared with PMCS, Cu/PMCS killed bacteria more quickly and effectively, thus facilitating wound healing owing to the enhanced photothermal effect and slow release of Cu^2+^. Cu/PMCS promoted cell migration and angiogenesis and upregulated the expression of related genes to accelerate wound healing. Cu/PMCS has potential applications in treating melanoma and repairing wounds with its antitumor, antibacterial, and wound‐healing properties.

## Graphical abstract

A Cu‐Zn bimetallic single‐atom (Cu/PMCS) is constructed with augmented photothermal effect and catalytic activity due to the localized surface plasmon resonance (LSPR) effect caused by copper doping, which provides a new strategy for improving photothermal and chemodynamic synergistic therapy. Additionally, Cu‐PMCS with anti‐tumor, anti‐bacterial, and wound‐healing properties, is expected to meet the dual clinical needs of melanoma treatment and repair.

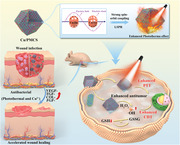

## 1 Introduction

Cutaneous melanoma is one of the most aggressive skin cancers, with high morbidity and mortality rates.^[^ ^1^ ^]^ Currently, surgical resection combined with radiotherapy or chemotherapy is the most common treatment for cutaneous melanoma.^[^ ^2^ ^]^ However, with surgical resection, cancer recurs easily. Additionally, wound infection causes further damage, making the resulting wound difficult to heal.^[^ ^3^ ^]^ Moreover, traditional chemotherapy and radiotherapy may cause drug resistance and damage normal tissues, causing irreversible harm to the patient.^[^ ^4^ ^]^ Therefore, developing multifunctional nanomaterials with anticancer, antibacterial, and wound‐healing properties is vital for treating epidermal cutaneous melanoma. Nanomaterials with photothermal combined chemodynamic therapy (PTT‐CDT) have attracted the attention of researchers owing to their excellent synergistic therapeutic effects.^[^ ^5^ ^]^ Notably, increasing the local temperature at the tumor site effectively improves the catalytic efficiency of Fenton reagent.^[^ ^6^ ^]^ Consequently, the preparation of multifunctional materials with higher photothermal conversion efficiency and catalytic activity can achieve better synergistic therapeutic effects in cutaneous melanoma.

Recently, plasmonic materials have attracted considerable attention for their superior photocatalytic and photothermal effects due to their strong light absorption and conversion ability via localized surface plasmon resonance (LSPR).^[^ ^7^ ^]^ Free electrons with high energy in plasmonic materials oscillate collectively by exciting LSPR under light irradiation, which could enhance catalytic activity. Additionally, the lattice temperature increases via electron‐phonon scattering, leading to a local heating effect of the plasmonic materials.^[^ ^8^ ^]^ Precious metals have been proven to exhibit good LSPR effects.^[^ ^8^, ^9^ ^]^ For example, Tao et al. synthesized multibranched gold nanocomposites with significant plasmon resonance in the near‐infrared (NIR)‐II window and controlled CRISPR‐Cas9 delivery for synergistic gene‐photothermal tumor therapy.^[^ ^10^ ^]^ Moreover, previous reports confirmed that metal nanostructures, including gold nanoparticles and palladium‐ or platinum‐coated gold nanorods, exhibit plasmonically enhanced peroxidase‐like activity under plasmonic excitation in the range of visible light to NIR.^[^ ^9^, ^11^ ^]^ Additionally, all forms of plasma metals are theoretically capable of producing LSPR, whether the plasmonic metal is present as metal ions, metal singlets, or metal single atoms in nanomaterials.^[^ ^12^ ^]^ However, plasma catalysis is still mainly used in electrocatalysis, photocatalysis, and gas‐phase catalysis, with little research on biological enzyme‐like catalysis. Additionally, the high cost of precious metals limits their biological applications.

Reassuringly, researchers have found that nonprecious metals, especially Cu‐based nanomaterials, also exhibit excellent LSPR effects and possess modulated plasma properties, such as resonance wavelength and LSPR intensity, through the adjustment of their chemical composition and structure;^[^ ^13^ ^]^ the most common being metal doping, where different metals possess different resonant photon wavelengths. The construction of Cu‐based bimetals and poly metals can change the position of the absorption peak and further expand the spectral response range. Additionally, the electronic structure, such as the d‐band center, can be altered by the electronic interactions between different metals, affecting the LSPR intensity.^[^ ^7^, ^14^ ^]^ Besides, redox‐active Cu‐based nanomaterials can effectively deplete overexpressed reductive glutathione (GSH) and catalyze the generation of highly oxidative hydroxyl radicals (·OH) from local H_2_O_2_ in the tumor microenvironment.^[^ ^15^ ^]^ Under light irradiation, Cu‐based nanomaterials generate hot electrons and holes through the LSPR effect, which may further enhance the GSH depletion capacity and Fenton‐like catalytic reaction rate, enabling the catalytic treatment of tumors. Furthermore, the biological effects of Cu^2+^ are closely related to their concentration; at appropriate concentrations, they are effective in killing bacteria and promoting vascularization, which accelerates wound healing.^[^ ^16^ ^]^ However, excess concentrations will inevitably be toxic to normal cells.^[^ ^17^ ^]^ Currently, the plasma catalysis of Cu‐based nanomaterials based on the LSPR effect is still focused on industrial catalysis with high content of copper. Improving copper utilization by reducing its amount in Cu‐based nanomaterials with LSPR effects for biological applications is challenging.

Recently, single‐atom catalysts (SACs) have attracted the attention of researchers due to their excellent catalytic efficiency. A series of highly active SACs with well‐defined metal‐nitrogen‐carbon coordination structures (M–N–C, M = Fe, Co, Zn, and Cu) have been developed. As expected, it maximizes the use of metal ions and effectively reduces their side effects.^[^ ^18^ ^]^ Among them, ZIF‐8‐derived porphyrin‐like structures (PMCS) with zinc centers have been used as a special nanoplatform for the construction of SACs with M‐N‐C structures owing to their good biosafety and excellent photothermal and catalytic properties and have made some progress in biomedical fields, due to its antibacterial, anti‐inflammatory, and antitumor properties.^[^ ^12^, ^19^ ^]^ Zhao et al. recently found that PMCS‐derived Co–Fe bimetallic single‐atom have a superior catalytic activity than that of monometallic single‐atom, achieving efficient treatment with very low metal concentrations.^[^ ^20^ ^]^ Consequently, constructing PMCS‐derived Cu‐based single‐atom catalyzing photothermal agents with LSPR effect may achieve excellent synergistic PTT‐CDT antitumor effects with better biosafety. Although there are many reports on single atoms, there are fewer reports on the LSPR effect on the photothermal properties and enzymatic catalytic activity of the ZIF‐8‐derived single atoms.

**Scheme 1:**
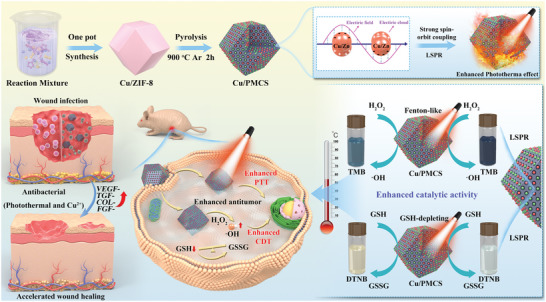
Schematic illustration of the synthesis process and therapeutic effects of Cu/PMCS. Cu/PMCS exhibits augmented photothermal effect due to the strong spin‐orbit coupling and localized surface plasmon resonance (LSPR) effect. The increased temperature and LSPR further enhance the catalytic activity in the Fenton‐like reaction and glutathione depletion capacity of Cu/PMCS. Therefore, Cu/PMCS performs better synergistic anti‐melanoma effects. Cu/PMCS is also effective in promoting the healing of infected wounds.

Based on the above considerations, we designed a Cu‐Zn bimetallic single‐atom (Cu/PMCS) to improve the photothermal performance, catalytic activity, and GSH depletion capacity of PMCS using the LSPR effect to achieve better PTT synergy with CDT for cutaneous melanoma treatment. First, Cu/PMCS was synthesized by a two‐step hydrothermal pyrolysis method, the photothermal properties of copper doping on PMCS were systematically investigated, and the related mechanism was revealed using DFT theoretical calculations. The effects of Cu doping and NIR on the catalytic activity and GSH depletion ability of PMCS were further investigated, and the related mechanisms were explained. Finally, the effect of copper doping on the antitumor and antibacterial abilities of PMCS in promoting wound healing was investigated in vitro and in vivo. In conclusion, this study not only increased the photothermal conversion efficiency of photothermal agents and the catalytic effect of the Fenton reagent, but also improved Cu^2+^ utilization by reducing its amount in Cu‐based nanomaterials with LSPR effects for biological applications. Furthermore, it provides a theoretical basis for the NIR‐induced LSPR effect of Cu‐based nanomaterials to enhance their photothermal properties and catalytic activity but also demonstrates that Cu/PMCS has a promising future in the postoperative treatment and repair of cutaneous melanoma (**Scheme** **1**).

## 2 Results and Discussion

### 2.1 Synthesis and Characterization of Cu/PMCS

**Figure 1:**
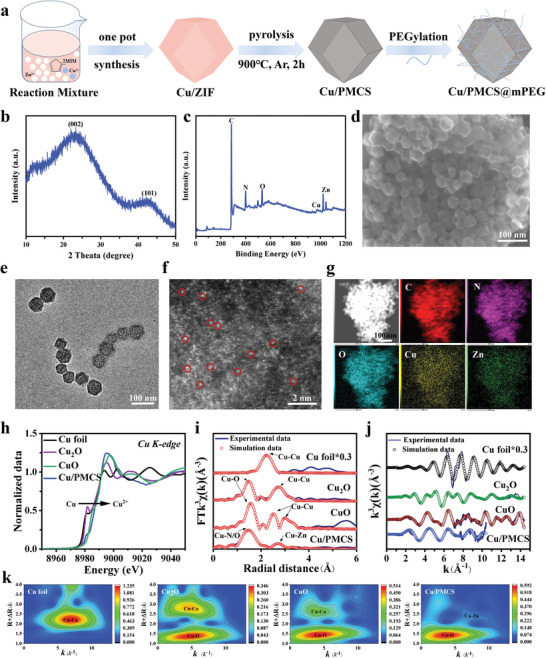
Synthesis and characterization. a) Schematic diagram of the Cu/PMCS synthesis process. b) XRD patterns, c) XPS full spectra, d) SEM image, e) TEM image, f) AC‐HAADF‐STEM image (bright white dots, highlighted by red circles), g) HAADF‐STEM image and EDS mapping images of Cu/PMCS. h) XANES spectra. i) Fourier transformations in R‐space and j) K‐space. k) Wavelet transform (WT) of Cu foil, Cu_2_O, CuO, and Cu/PMCS.

Cu/PMCS was synthesized according to the scheme shown in **Figure** **1**a. Cu‐doped ZIF‐8 (Cu‐Zn bimetallic organic framework, denoted as Cu/ZIF) was first synthesized by a one‐step method, and X‐ray diffraction (XRD) patterns and transmission electron microscopy (TEM) images confirmed the successful preparation of Cu/ZIF (Figure S1, Supporting Information). Subsequently, Cu/ZIF was further pyrolyzed under an Ar atmosphere to obtain Cu/Zn single‐atom (Cu/PMCS) with an M‐N‐C structure. PMCS without Cu doping was also synthesized by the same method. The XRD pattern of Cu/PMCS (Figure 1b) shows no crystalline peaks corresponding to Cu, Zn, or their chemical compounds; only broad peaks in the (002) and (101) face of graphite were detected at ≈25° and 43°. Meanwhile, the X‐ray photoelectron spectroscopy (XPS) full spectra of Cu/PMCS at Cu 2p (Figure 1c) indicated the successful doping of Cu. The scanning electron microscopy (SEM) (Figure 1d) and TEM images (Figure 1e) show that the particle size of Cu/PMCS was ≈50 nm. Additionally, TEM reveals the morphology of Cu/PMCS, which retained the polyhedral morphology of Cu/ZIF (Figure S1b, Supporting Information) after calcination and had a porous structure. The DLS test showed that the particle size distribution of Cu/PMCS was relatively uniform and well‐dispersed (Figure S2, Supporting Information). Moreover, the aberration‐corrected high‐angle annular dark‐field scanning transmission electron microscopy (AC‐HAADF‐STEM) image (Figure 1f) shows the absence of clusters and particles in Cu/PMCS and the presence of visible individual atoms (bright white dots, highlighted by red circles) loaded on N‐doped carbon matrix carriers, which could be Zn, Cu, or Zn/Cu dual single‐atom. The visible individual atoms representing Zn single‐atom are shown in the AC HAADF‐STEM image of the PMCS. Furthermore, the energy‐dispersive X‐ray spectroscopy (EDS) mapping (Figure 1g) showed that C, N, O, Zn, and Cu were uniformly distributed in the sample. Lastly, mPEG‐DSPE (5 kD) was used to modify Cu/PMCS to obtain good physiological stability. The zeta potential of PEGylated Cu/PMCS changed from −15 to −10 mV (Figure S3, Supporting Information), demonstrating the successful modification with mPEG‐DSPE.^[^ ^19a^ ^]^

The coordination structures of individual Cu and Zn atom in the Cu/PMCS were further characterized by synchrotron X‐ray absorption near‐edge structure (XANES) and extended X‐ray absorption fine structure (EXAFS). The Cu K‐edge XANES (Figure 1h) spectra demonstrated that the Cu K‐edge distribution of Cu/PMCS was between that of Cu_2_O and CuO, closer to that of CuO, indicating the presence of a portion of the positive charge in Cu/PMCS with an oxidation state between +1 and +2, closer to +2. As shown in Figure 1i, the FT‐EXAFS spectrum of Cu/PMCS showed a dominant peak at ≈1.53 Å, which is attributed to Cu–N(C) coordination.^[^ ^12a^ ^]^ Moreover, no Cu‐Cu coordination signal was detected in Cu/PMCS, suggesting that Cu atom existed as isolated single atomic sites. The coordination parameters further fitted using the synchrotron radiation data (Table S1, Supporting Information) showed that the coordination environment of Cu in Cu/PMCS was a Cu–N/O hexa‐ligand configuration, similar to the planar tetra‐ligand N and axial di‐ligand O. The presence of O species may be due to the contribution of oxygen or water vapor. The fitted curves of EXAFS in the R‐space (Figure 1i) and K‐space (Figure 1j) were similar to those of EXAFS. The good fit of the experimental curves indicates the accuracy and reliability of the above results. Furthermore, the wavelet transform (WT) map (Figure 1k) of Co/PMCS showed that there was only one WT with a maximum value of 4 Å^‐1^ in k‐space compared to Cu foil, Cu_2_O, and CuO, which was consistent with Cu‐N bonding, further confirming the presence of Cu in Cu/PMCS centered in atomically dispersed mononuclear Cu and coordinated to N atoms. Similarly, the AC‐HAADF‐STEM image (Figure S4, Supporting Information), XANES spectra of the Zn K‐edges (Figure S5a, Supporting Information), EXAFS (Figure S5b‐c, Supporting Information), coordination parameters (Table S1, Supporting Information), and WT (Figure S5d, Supporting Information) indicate the presence of Zn in Cu/PMCS and PMCS in the form of single‐atom. The coordination environment of Cu in Cu/PMCS was a Cu–N/O hexa‐ligand configuration, similar to the planar tetra‐ligand N and axial di‐ligand O. And the coordination environment of Zn in Cu/PMCS was also a Zn‐N/O hexa‐ligand configuration. The presence of O species may be due to the contribution of oxygen or water vapor. Additionally, a weak Cu–Zn signal was detected in the outer shell layer, indicating that the Cu–Zn bond present in the Cu/PMCS is much low and is not sufficient to affect the overall structure of the material. Therefore, it can be assumed that almost all of the Cu/PMCS are single atoms. Thus, a bimetal single‐atom Cu/PMCS was successfully obtained using a two‐step method.

To further reveal the effect of Cu doping on the enzymatic activity and photothermal properties of PMCS, PMCS, Cu/PMCS‐1, Cu/PMCS‐2, and Cu/PMCS‐3 doped with different concentrations of Cu were synthesized. ZIF, Cu/ZIF‐1, Cu/ZIF‐2, and Cu/ZIF‐3 doped with different concentrations of Cu first were synthesized. From their XRD patterns (Figure S6a, Supporting Information) and TEM images (Figure S6b, Supporting Information), no significant changes were observed in the crystallinity and morphology of the ZIF after Cu doping. The above materials were then heat‐treated, and their XRD patterns and TEM images confirmed the successful preparation of the PMCS, Cu/PMCS‐1, and Cu/PMCS‐2 (Cu/PMCS) after pyrolysis (Figure S7a‐b, Supporting Information). The full XPS spectrum (Figure S7c, Supporting Information) revealed their main composition to be carbon, nitrogen, and oxygen, with the percentages of each atom shown in Table S2, Supporting Information. Additionally, the Cu 2p XPS spectra (Figure S7d, Supporting Information) and semi‐quantitative results indicate that the Cu content increased sequentially from PMCS, Cu/PMCS‐1, and Cu/PMCS‐2 to Cu/PMCS‐3. Next, the metal loadings of the different samples were further quantified using inductively coupled plasma emission spectroscopy (ICP‐OES) (Table S3, Supporting Information), and the Cu concentrations of PMCS, Cu/PMCS‐1, Cu/PMCS‐2, and Cu/PMCS‐3 were 0 wt.%, 0.22 wt.%, 0.6 wt.%, and 1.4 wt.%, while the Zn concentrations of PMCS, Cu/PMCS‐1, Cu/PMCS‐2, and Cu/PMCS‐3 were 6.23 wt.% 6.08 wt.% 4.06 wt.% and 3.09 wt.%. The AC‐HAADF‐STEM images show that the metals are in the single atom state in both PMCS, Cu/PMCS‐1 Cu/PMCS‐2, and Cu/PMCS‐3 (Figure S8, Supporting Information). The above characterization confirmed the successful preparation of PMCS, Cu/PMCS‐1, Cu/PMCS‐2, and Cu/PMCS‐3 with different Cu concentrations. Unless otherwise stated, Cu/PMCS refers to Cu/PMCS‐2.

### 2.2 Effect of Cu Doping on the In Vitro Photothermal Performance of PMCS and DFT Theoretical Calculation

**Figure 2:**
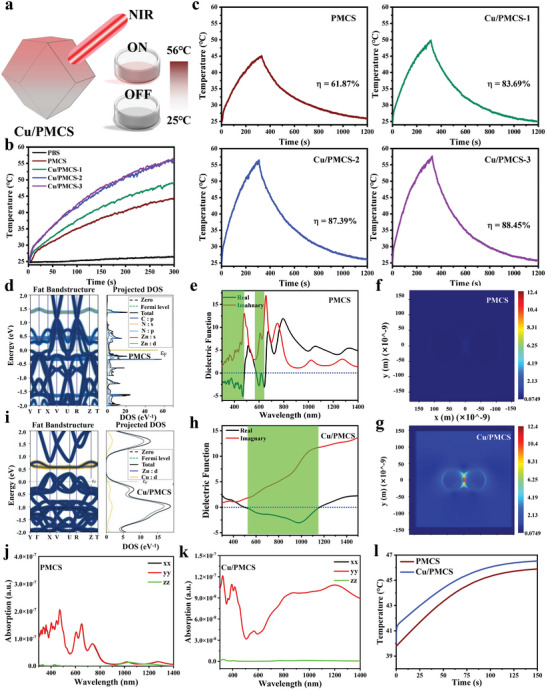
In vitro photothermal properties and theoretical calculations. a) Schematic diagram of photothermal heating of Cu/PMCS. b) Heating curves of under 808 nm NIR irradiation with power density of 0.7 W cm^−2^ for 5 min, and c) Photothermal transition efficiencies of PMCS, Cu/PMCS‐1, Cu/PMCS‐2, and Cu/PMCS‐3 dispersed in PBS. Energy bands and density of states (DOS) of d) PMCS and i) Cu/PMCS. Dielectric constants of e) PMCS and h) Cu/PMCS. Thermal field maps of plasmons f) PMCS and g) Cu/PMCS. Absorption spectra at 808 nm of j) PMCS and k) Cu/PMCS; l) Heating curve under 808 nm NIR irradiation with power density of 1 W cm^−2^ for 150 s plotted by simulation. (Cu/PMCS refers to Cu/PMCS‐2)

To test the in vitro photothermal performance of the PMCS, Cu/PMCS‐1, Cu/PMCS‐2, and Cu/PMCS‐3, an 808 nm laser (0.7 W cm^−2^) was used for 5 min, and the temperature change was recorded (**Figure** **2**a). The heating curves (Figure 2b and Figure S9, Supporting Information) showed that the photothermal ability became stronger as the Cu concentration increased; Cu/PMCS‐2 and Cu/PMCS‐3 rapidly warmed up to ≈56 °C within 5 min. We further calculated the photothermal transition efficiencies of the different samples, as shown in Figure S10, Supporting Information and Figure 2c, including, 61.87%, 83.69%, 87.39%, and 88.45% for PMCS, Cu/PMCS‐1, Cu/PMCS‐2, and Cu/PMCS‐3, respectively. This indicates that the photothermal transition efficiency increased significantly with increasing Cu concentration. Additionally, the heating curves of different concentrations of Cu/PMCS and those of Cu/PMCS at different powers were analyzed. The temperature increase of Cu/PMCS was concentration‐dependent (Figure S11, Supporting Information), which could be controlled by adjusting the laser power (Figure S12, Supporting Information); additionally, Cu/PMCS exhibited good photothermal stability (Figure S13, Supporting Information). The LSPR of Cu may improve the photothermal effect of the material; however, the LSPR intensity and resonance wavelength were influenced by various factors, such as the type of material, structure, and size. Therefore, to systematically explain why Cu doping could enhance the photothermal performance of PMCS, DFT theoretical calculations were used to reveal the relevant mechanism underlying photothermal enhancement.

From the energy band and density of the state plots of PMCS (Figure 2d) and Cu/PMCS (Figure 2i), it is observed that the stronger photothermal effect of Cu/PMCS compared with that of PMCS was due to Cu doping. This introduced spin‐orbit coupling, resulting in the d‐orbital transition of Cu and Zn not being released by emission and the release of energy in the form of inter‐system scramble crossing (source of the photothermal theory).^[^ ^21^ ^]^ The addition of Cu impurity energy levels induced a stronger dielectrophoretic absorption, which may lead to a negative dielectric function according to the Kramer–Kronig relation, inducing LSPR.^[^ ^22^ ^]^ Therefore, the dielectric constant of the complex was calculated, and it is found that the band of the PMCS with possible plasmons shifted to the right and became wider after Cu doping (Figure 2e,h), suggesting that Cu/PMCS may produce plasmons at 808 nm, whereas PMCS cannot. The mode field of the plasmons produced by Cu/PMCS is shown in Figure 2g, and a strong field is always present in the interparticle region, which is the hot spot formed by the intersphere LSPR. However, for the PMCS, the dielectric function was positive at 808 nm, so it could not induce plasmons. Therefore, there was no equipartition excitation electric field pattern (Figure 2f). Furthermore, the absorption spectra and heating curves of the PMCS and Cu/PMCS were simulated and calculated using the finite element method. As shown in the absorption spectra (Figure 2j‐k), Cu/PMCS exhibited significant absorption at 808 nm, whereas PMCS had no absorption. This explains the significantly higher heating of the Cu/PMCS than that of the PMCS (Figure 2l). The above analysis reveals that the stronger photothermal effect of Cu/PMCS compared with that of PMCS was due to the introduction of impurity energy levels via Cu doping, causing a new d‐orbital transition with strong spin‐orbit coupling. Lastly, the negative dielectric effect generated by the transition‐induced LSPR enhanced the photothermal effect.

In addition, the effect of carbonization temperature on the photothermal properties was tested. The results showed that the photothermal properties were enhanced with increasing temperature, but 900°C was the best, which may be related to the light absorption of Cu/PMCS at 808 nm (Figure S14, Supporting Information).^[^ ^19a^ ^]^

### 2.3 Effect of Cu Doping on the Catalytic Activity of PMCS and the Related Mechanism

**Figure 3:**
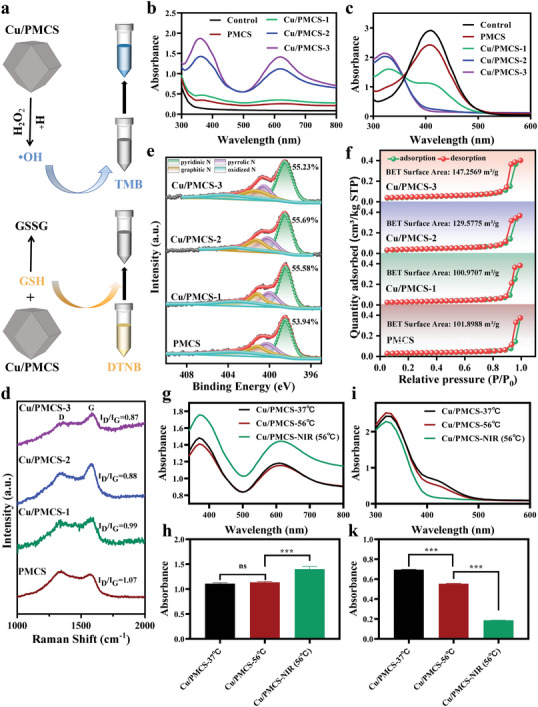
Catalytic activity and GSH depletion capacity. a) Schematic diagram of the principle of peroxidase activity (above) and GSH depletion capacity test (below). b) The absorbance spectra of TMB for detecting the ability of PMCS, Cu/PMCS‐1, Cu/PMCS‐2, Cu/PMCS‐3 to produce ·OH (pH = 6.5, H_2_O_2_:10 mM). c) The absorbance spectra of DTNB for detecting GSH (0.25 mM) consumption of PMCS, Cu/PMCS‐1, Cu/PMCS‐2, Cu/PMCS‐3. d) The Raman spectra. e) XPS N1s spectra. f) Nitrogen sorption isotherms of PMCS, Cu/PMCS‐1, Cu/PMCS‐2, Cu/PMCS‐3. g) Qualitative and h) Quantitative analysis of the ability to produce ·OH (pH = 6.5, H_2_O_2_:10 mM). i) Qualitative and k) Quantitative analysis of GSH (0.25 mM) consumption by Cu/PMCS at 37 °C and 56 °C and by Cu/PMCS at 56 °C under 808 nm NIR irradiation with power density of 0.7 W cm^−2^ for 5 min. (Cu/PMCS refers to Cu/PMCS‐2) Data represent means±SD (*n*  = 3). Statistical significance was calculated by one‐way ANOVA analysis. *p < 0.05; **p < 0.01; ***p < 0.001.

Next, the catalytic activity and ability to deplete GSH of different samples (**Figure** **3**a) were tested in vitro. First, the ability of PMCS, Cu/PMCS‐1, Cu/PMCS‐2, and Cu/PMCS‐3 to catalyze the production of ·OH from H_2_O_2_ was evaluated, using H_2_O_2_ and 3,3′,5, 5′‐tetramethylbenzidine (TMB) as substrates. The absorbance value of the material after reaction with TMB reflects the ability of the material to catalyze the production of ·OH from H_2_O_2_. The larger the absorbance value, the stronger the ability to produce ·OH, which changes the TMB solution from colorless to blue. Figure 3b and Figure S15, Supporting Information, show that the ability to produce ·OH became stronger with increasing Cu concentration, indicating enhanced catalytic activity in the Fenton‐like reaction. In addition, it is found that the higher the concentration of the material, the greater the ability to produce ·OH (Figure S16, Supporting Information). The catalytic efficiency of Cu/PMCS was significantly higher under acidic conditions (Figure S17, Supporting Information), and its catalytic activity was dependent on H_2_O_2_ concentration (Figure S18, Supporting Information). High expression of GSH at the tumor site depletes ·OH; therefore, GSH depletion may also improve the effect of tumor catalytic therapy. The ability of PMCS, Cu/PMCS‐1, Cu/PMCS‐2, and Cu/PMCS‐3 to deplete glutathione was further evaluated, using the 5,5′‐dithiobis (2‐nitrobenzoic acid) (DTNB) indicator. As shown in Figure 2c and Figure S19, Supporting Information, the characteristic peak of the product of DTNB and GSH at ≈412 nm significantly decreased with increasing Cu concentration, indicating enhanced GSH depletion. Additionally, it was found that the higher the concentration of material, the greater the depletion level (Figure S20, Supporting Information). In summary, the catalytic activity in the Fenton‐like reaction and the GSH depletion capacity of PMCS became stronger with increasing Cu concentration. The Cu monatomic content PMCS, Cu/PMCS‐1, Cu/PMCS‐2, and Cu/PMCS‐3 is increased and the Zn monatomic content showed a decrease, with an increase in ROS production and GSH consumption, which suggests that Cu single atoms in Cu/PMCS has a greater effect on enzyme activity than Zn single atoms in Cu/PMCS.

The catalytic activity of PMCS may be related to the C defect, metal active site, and specific surface area of the material.^[^ ^12^, ^19^, ^20^, ^23^ ^]^ Thus, Raman, XPS, and Brunner−Emmet−Teller (BET) tests were performed to investigate the enhancing effect of Cu doping on the PMCS catalytic activity. The Raman spectra of PMCS, Cu/PMCS‐1, Cu/PMCS‐2, and Cu/PMCS‐3 (Figure 2d) showed two typical peaks corresponding to graphitic carbon at ≈1340 cm^−1^ and 1575 cm^−1^, where the D band (1340 cm^−1^) corresponds to disordered carbon or defective graphitic structures and the G band (1575 cm^−1^) is typical for graphite.^[^ ^19a^ ^]^ The *I*_D_/*I*_G_ ratios of PMCS, Cu/PMCS‐1, Cu/PMCS‐2, and Cu/PMCS‐3 were 1.07, 0.99, 0.88, and 0.86, respectively, which indicates that the ratio of *I*_D_/*I*_G_ progressively decreased as the Cu concentration increased, suggesting that the doping of copper results in fewer topological defects in PMCS. The N 1s XPS spectra (Figure 2e) indicate that four types of nitrogen are present in PMCS, Cu/PMCS‐1, Cu/PMCS‐2, and Cu/PMCS‐3, including the pyridine state (398.5 eV), pyrrole state (400.1 eV), graphitic state (401.4 eV), and weakly oxidized nitrogen (404.3 eV), respectively. Their contents in the different samples are listed in Table S4, Supporting Information. Furthermore, the binding of pyridinic nitrogen with Cu or Zn single‐atom increased with increasing Cu and decreasing Zn concentration, as well as a slight increase in pyridine N (Tables S3–S4, Supporting Information), indicating an increasing number of Cu active sites, which may improve the catalytic activity of Cu/PMCS. Meanwhile, the BET test results (Figure S21, Supporting Information, and Figure 2f) showed that with the increase in Cu concentration, the pore size decreased and the specific surface area increased significantly, facilitating the adsorption of the reactants and improving the catalytic activity. Additionally, the pore size decreased and the specific surface area increased indicated the topological defects in PMCS gradually decreased.^[^ ^24^ ^]^ Combined with the above analytical results, Cu doping enhanced the catalytic activity of PMCS, possibly because of increased Cu active sites and specific surface area.

Notably, we also tested the effects of the catalytic activity and GSH depletion capacity of Cu/PMCS with and without NIR (808 nm, 0.7 W cm^−2^, 5 min) irradiation or at different temperatures (37 °C and 56 °C). We found that at low H_2_O_2_ concentrations (0.1 mM), the catalytic activity was significantly higher at 56 °C than at 37 °C, indicating that elevated temperatures increase the catalytic activity of Cu/PMCS (Figure S22a,c, Supporting Information). However, there was no significant difference in the catalytic activity of Cu/PMCS (Figure S22b,d, Supporting Information, and Figure 2f‐h) at high H_2_O_2_ concentrations (1 mM and 10 mM), which may be due to the different catalytic rates at different concentrations. Notably, NIR light irradiation further enhanced the catalytic activity of the Cu/PMCS at both low and high H_2_O_2_ concentrations. Additionally, increasing the temperature enhanced the GSH depletion capacity of the Cu/PMCS at different GSH concentrations. Similarly, the GSH depletion capacity of the Cu/PMCS was further enhanced by NIR light irradiation (Figure S23, Supporting Information, and Figure 2i‐k). Plasma metals are reportedly capable of plasma catalysis through LSPR; plasma catalysis can further improve the catalytic effect beyond the equilibrium limit of thermal catalytic reactions. Cu nanomaterials exhibit excellent LSPR effects in the UV‐visible and NIR ranges and are a good platform for efficient light‐driven chemical reactions. This suggests that further enhancement of the catalytic activity and GSH depletion capacity of Cu/PMCS under NIR irradiation may be due to the LSPR effect of Cu/PMCS, which was confirmed through DFT calculations.

Also, the effects of carbonization temperature on the ·OH generation were also evaluated. As shown in Figure S24a, the ·OH generation also increased gradually with the increase of temperature, among which 800 °C and 900 °C were relatively better than 1000 °C, which caused by more metal evaporation at high temperatures.^[^ ^19b^ ^]^ In addition, Figure S24b, Supporting Information, showed that GSH is consumed more with increasing temperature, where 800 °C, 900 °C, and 1000 °C are almost the same.

### 2.4 Effect of Cu Doping on the In Vitro Anti‐Melanoma Ability of PMCS

**Figure 4:**
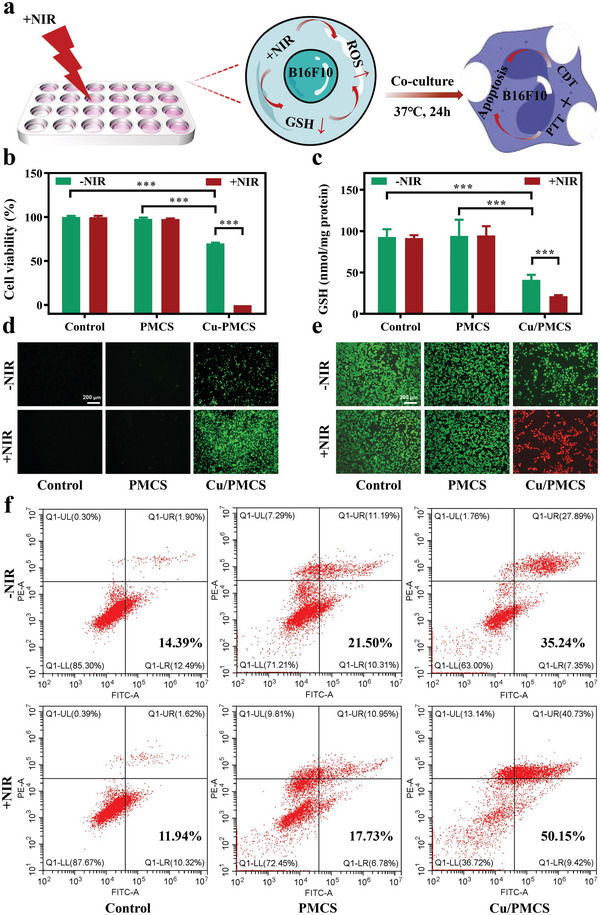
The ability to synergistically inhibit melanoma cells in vitro. a) Schematic diagram of in vitro antitumor assay under 808 nm NIR irradiation with power density of 0.7 W cm^−2^ for 5 min. b) Viability of B16F10 cells. c) GSH content in B16F10 cells. d) ROS staining (green: ROS) and e) live‐dead staining of B16F10 cells (green: lived cells, red: died cells). f) Flow cytometry analysis of the effects of different formulations on B16F10 cell apoptosis (the two right quadrants represent apoptosis). Data represent means±SD (*n*  = 4). Statistical significance was calculated by two‐way ANOVA analysis. *p < 0.05; **p < 0.01; ***p < 0.001.

Encouraged by the excellent catalytic activity and photothermal properties of Cu/PMCS, the inhibitory effects of PMCS and Cu/PMCS on mouse melanoma cells (B16F10) were first evaluated at the cellular level (**Figure** **4**a). As shown in Figure S25, Supporting Information, the higher the power, the lower the cell viability, and the more pronounced the inhibitory effect. The cell viability was ≈53% when the power was 0.7 W cm^−2^, which was used for subsequent experiments. From the cell proliferation results (Figure 4b), only Cu/PMCS showed an antitumor effect when no NIR was applied, indicating that Cu doping enhanced the antitumor effect of PMCS in vitro. When NIR was applied, we observed no significant change in the control and PMCS groups compared with the NIR group; however, the cell viability of Cu/PMCS was approximately zero, which was due to the good photothermal effect of Cu/PMCS, and further confirmed that Cu doping could enhance the photothermal effect of PMCS. Notably, the inhibition rate of Cu/PMCS on B16F10 cells was ≈47% when only PTT treatment was available (0.7 W cm^−2^). When only CDT treatment was available (‐NIR), the inhibition rate was ≈30%; however, when PTT combined with CDT treatment was available (+NIR), Cu/PMCS inhibited B16F10 cells by ≈100%, indicating that PTT enhanced the therapeutic effect of CDT. On the other hand, the inhibition rate of Cu/PMCS is higher than PMCS (0%), showing that Cu doping could enhance the CDT of PMCS. To verify these findings, we further tested the ability of PMCS and Cu/PMCS to scavenge GSH (Figure 4c) and produce ROS (Figure 4d) in B16F10 cells. Cu doping significantly enhanced the intracellular GSH scavenging and ROS‐producing abilities of PMCS, consistent with the in vitro enzyme activity test results. Moreover, the ability of Cu/PMCS to scavenge GSH and produce ROS significantly increased after the addition of NIR, suggesting that PTT enhanced GSH consumption and ROS production by Cu/PMCS, which coincidentally explained the previous cell proliferation results. Besides cell proliferation, the inhibitory effect of PMCS and Cu/PMCS on B16F10 cells was further evaluated using live‐dead staining and flow cytometry. The treated cells were fluorescently stained using live‐dead cell stains. The results (Figure 4e) were consistent with the cell proliferation results (green fluorescence for live cells and red fluorescence for dead cells). Apoptosis and the cell cycle were detected via flow cytometry. As shown in Figure 4f, the apoptosis rate induced by Cu/PMCS was higher than that by PMCS, and the apoptosis rate of the Cu/PMCS+NIR group was even higher than 50.15%, indicating that the apoptosis rate induced by the CDT/PTT synergistic treatment was significantly higher and that the synergistic treatment was more effective. In addition, the cell cycle analysis results (Figure S26, Supporting Information) indicated that CDT/PTT mainly blocked the growth of the S‐phase cells. The above results suggest that Cu doping enhanced the CDT/PTT‐based synergistic inhibitory effect of PMCS on mouse melanoma cells (B16F10).

### 2.5 Effect of Cu Doping on the Effect of PMCS on Melanoma Treatment In Vivo

**Figure 5:**
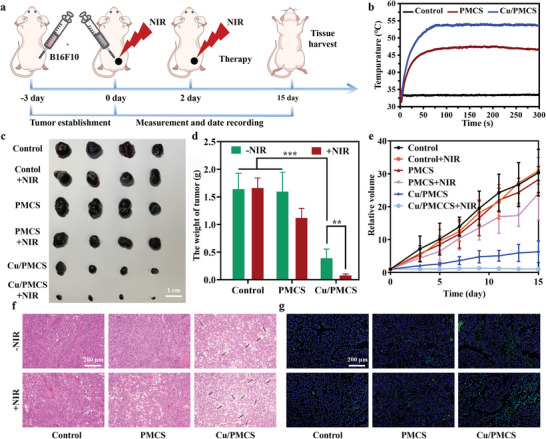
The synergistic treatment of melanoma in vivo. a) Schematic illustration of the injection process and timeline of cancer therapy under 808 nm NIR irradiation with power density of 0.7 W cm^−2^ for 5 min. b) The heating curves of the tumor sites in different groups of B16F10 tumor‐bearing mice. c) The isolated tumor photographs at Day 15. d) The weight of isolated tumors at Day 15. e) Relative volume curves after treatment with the various formulations. f) HE staining (black arrows: necrosis) and g) TUNEL staining of tumors treated with various formulations (green: apoptosis). Data represent means ± SD (*n*  = 4). Statistical significance was calculated by two‐way ANOVA analysis. *p < 0.05; **p < 0.01; ***p < 0.001.

Based on the above in vitro cellular results, the effect of Cu doping on PMCS in melanoma treatment was further investigated in vivo by subcutaneously injecting B1610 cells into BALB/c nude mice to establish a mouse subcutaneous melanoma model (**Figure** **5**a). As cutaneous melanoma is in the superficial tissue of the body, the nanomaterials were intratumorally injected into tumor sites to improve the treatment by increasing drug accumulation effect and reduce the risk of systemic toxicity.^[^ ^25^ ^]^ First, the heating curves of PBS (control), PMCS, and Cu/PMCS at the tumor site in the mice were tested. Consistent with in vitro studies results, Cu doping significantly improved the photothermal effect of PMCS, and Cu/PMCS warmed up to 53.7 °C within 5 min (Figure 5b and Figure S27, Supporting Information). To evaluate the tumor‐suppressive effect of the material in vivo, the changes in tumors in mice during the 15 days of treatment were recorded, and all the mice were executed on Day 15 to prevent tumors exceeding the size of the animal test ethics. Photographs of mice after 15 days of receiving different treatments, isolated tumors, and the mass of isolated tumors are shown in Figure S28, Supporting Information, and Figure 5c‐d, respectively. The Cu/PMCS group had a significant inhibitory effect on melanoma compared with the PMCS group due to CDT treatment. Notably, Cu/PMCS almost completely inhibited melanoma growth in the presence of NIR, indicating that Cu/PMCS has an excellent CDT/PTT‐based synergistic anti‐melanoma effect. The relative volume changes in tumors in the different groups further indicate that PMCS significantly inhibited melanoma growth after Cu doping (Figure 5e). Additionally, HE staining and TUNEL staining of tumor sections showed that the control and PMCS groups maintained normal morphology. In contrast, the Cu/PMCS group showed partial necrosis and apoptosis. The Cu/PMCS+NIR group exhibited severely damaged tumor tissue, causing more extensive apoptosis and necrosis than the other groups (Figure 5f,g), which further proved by the Quantitative analysis of TUNEL staining (Figure S29, Supporting Information). Additionally, recurrence had not occurred in Cu/PMCS+NIR group during the treatment period. In summary, Cu doping enhanced the synergistic CDT/PTT‐based inhibitory effect of PMCS on mouse melanoma cells. Lastly, the HE staining results of the major organs of mice indicated the good biocompatibility of the material (Figure S30, Supporting Information).

### 2.6 Effect of Cu Doping on the In Vitro Antibacterial Effect of PMCS

**Figure 6:**
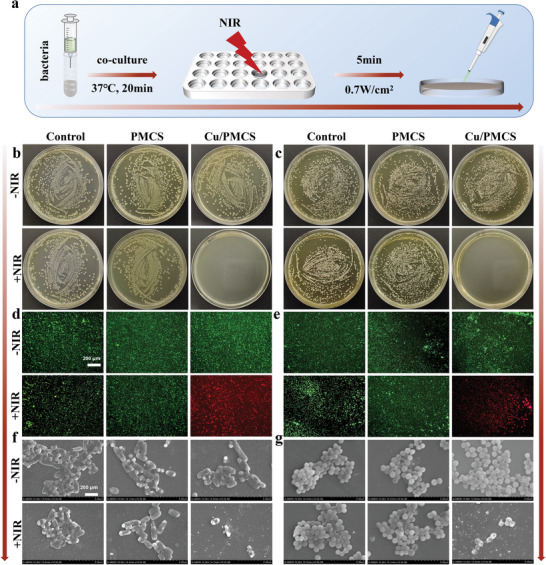
In vitro photothermal rapid antibacterial properties. a) Schematic diagram of in vitro photothermal rapid bactericidal antibacterial assay under 808 nm NIR irradiation with power density of 0.7 W cm^−2^ for 5 min. Bacterial colony plate images of b) *E. coli* and c) *S. aureus*. Live‐dead staining of d) *E. coli* and e) *S. aureus* (green: lived bacteria, red: died bacteria). Morphology of f) *E. coli* and g) S. aureus under Scanning electron microscope.

In addition to tumor recurrence, bacterial infection is a key clinical issue to be addressed after melanoma surgery. Therefore, the in vitro antibacterial efficacy of PMCS and Cu/PMCS against gram‐positive *Staphylococcus aureus* and gram‐negative *Escherichia coli* was evaluated, using bacterial colony counting, live‐dead staining, and morphological observation as characterization tools (**Figure** **6**a and Figure S31a, Supporting Information). First, the photothermal rapid bactericidal effects of PMCS and Cu/PMCS were evaluated (Figure 6b‐g). Photothermal fast sterilization means that the material was first cocultured with bacteria for 10 min, and then the antibacterial effect was directly evaluated after 5 min of NIR irradiation. The results of bacterial colony plate images (Figure 6b‐c), live‐dead staining (Figure 6d‐e), and morphology observation (Figure 6f‐g) all showed that Cu/PMCS had an excellent antibacterial effect after photothermal illumination, with an antibacterial rate of ≈100% compared with that in the control and PMCS groups, indicating that Cu doping could improve the photothermal‐based rapid bactericidal effect of PMCS.

Furthermore, compared to PMCS, Cu/PMCS had a significant and sustained antimicrobial effect at 24 h, which might be attributed to the release of Cu^2+^ from Cu/PMCS (Figure S31 and Table S5). The above results showed that Cu doping improved the photothermal rapid sterilization effect of PMCS and achieved sustained antibacterial activity by slowly releasing Cu ions, thus preventing bacterial reinfection effectively. The small amounts of copper release would not affect the photothermal performance, ·OH production, GSH consumption of Cu/PMCS, and biocompatibility (Figure S32, Supporting Information).

Finally, the antitumor effect of Cu/PMCS under bacterial‐tumor coexistence in vitro was evaluated by coculturing mouse melanoma cells (B16f10) with bacteria (*E. coli* and *S. aureus*) to simulate the occurrence of infection in the tumor part. The proliferation results (Figure S33, Supporting Information) showed that both Cu/PMCS and Cu/PMCS+NIR groups had significant inhibitory effects on tumor cells and bacteria compared with other groups, and Cu/PMCS+NIR had more than 90% inhibition of tumor cells and bacteria, which was consistent with the results of cells and bacteria cultured separately.

### 2.7 Biocompatibility and In Vitro Regenerative Capacity of Cu/PMCS

**Figure 7:**
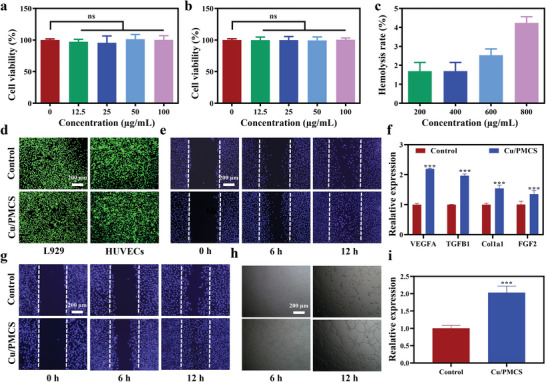
Biocompatibility and regeneration ability in vitro. The viability of a) L929 and b) HUVECs cocultured with Cu/PMCS for 24 h. c) Hemolysis rate of Cu/PMCS. d) Cell live‐dead staining of L929 and HUVECs cocultured with Cu/PMCS for 24 h (green: lived cells, red: died cells). e) Cell migration (purple: cell nucleus) and f) gene expression in L929. g) Cell migration, h) angiogenesis, and i) gene expression of VEGF in HUVECs. Data represent means ± SD (*n*  = 3). Statistical significance was calculated by one‐way ANOVA analysis and *t*‐test analysis. *p < 0.05; **p < 0.01; ***p < 0.001.

Good biocompatibility is the cornerstone of nanomaterials in clinical applications. Three typical skin cells [mouse epidermal fibroblasts (L929), mouse embryonic fibroblasts (NIH3T3), and human umbilical vein endothelial cells (HUVECs)] to were selected to evaluate the biocompatibility of Cu/PMCS in vitro. The cell proliferation results shown in **Figure** **7**a‐b and Figure S34a, Supporting Information, indicate that Cu/PMCS had good biocompatibility in vitro, confirmed by the results of the hemolysis assay (Figure 6c and Figure S35, Supporting Information) and cell live‐dead staining (Figure 6d and Figure S34b, Supporting Information). Additionally, the in vivo safety of Cu/PMCS was evaluated using BALB/c mice. The blood tests (Figure S36, Supporting Information) and HE staining of major organs (Figure S37, Supporting Information) indicate that Cu/PMCS exhibited good biocompatibility in vivo. Based on the good biocompatibility of Cu/PMCS, the regenerative ability of Cu/PMCS in vitro was further evaluated. A scratch assay was performed to investigate the effects of Cu/PMCS on cell migration. Figure 7e shows that Cu/PMCS could promote the migration of L929 cells, which is consistent with qualitative results (Figure S38a, Supporting Information). Cu/PMCS promoted the migration of L929 cells by upregulating the expression of pro‐wound healing factors, such as VEGFA, TGFB1, COLLA1, and FGF2 (Figure 7f). Additionally, Cu/PMCS upregulated the expression of the angiogenic factor VEGF to accelerate angiogenesis and promote the migration of HUVECs (Figure 7g‐i and Figure S38b‐c, Supporting Information). The above results suggest that Cu/PMCS has the potential to accelerate the wound healing process in vivo.

### 2.8 In Vivo Antimicrobial and Wound Healing

**Figure 8:**
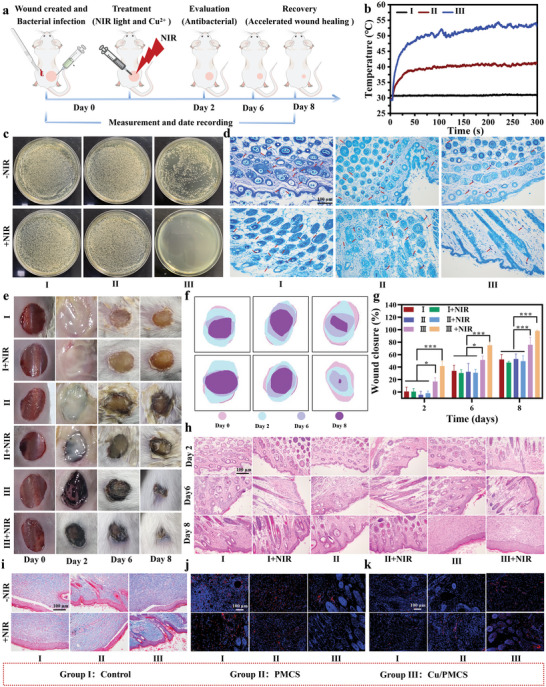
The antimicrobial and wound‐healing protocol in vivo. a) Treatment protocol for the infected full‐thickness wound‐healing model. b) Temperature change curves of the mouse wound sites under 808 nm NIR irradiation with power density of 0.7 W cm^−2^ for 5 min. c) Bacterial colony photos of the wounded skin. d) Giemsa staining of different groups (Deep purple: bacterial colonies). e) Wound photographs of the mice from Day 0 to Day 8 and f) the corresponding traces. g) The wound closure areas after various treatments at different time points. h) HE staining of the bacteria‐infected tissues after the different treatments. i) Masson staining (blue: Collagen fibers), j) CD68 staining (purple: DAPI, red: CD31), and k) CD31 staining (purple: DAPI, red: CD68) of different groups on Day 8. Data represent means ± SD (*n*  = 4). Statistical significance was calculated by two‐way ANOVA analysis. *p < 0.05; **p < 0.01; ***p < 0.001.

Encouraged by the excellent antibacterial ability and wound‐healing potential of Cu/PMCS in vitro, we further investigated these properties in vivo by establishing an infected whole‐layer wound‐healing model (**Figure** **8**a). The heating curve at the skin wounds of mice showed that the Cu/PMCS group heated significantly faster and higher than the PMCS group, further demonstrating that Cu doping could enhance the photothermal effect of PMCS (Figure 8b and Figure S39, Supporting Information). The in vivo bacterial colony counting (Figure 8c) and Giemsa staining (Figure 8d) results reconfirmed that Cu doping improved the photothermal‐Cu^2+^‐based synergistic antimicrobial effect of PMCS.

Bacterial infection is a key obstacle to wound healing, and a good antimicrobial capacity can effectively promote healing. After the infected full‐thickness wound models were established on the backs of the mice and treated, we recorded the healing of the wounds on the backs of the mice on days 2, 6, and 8. As shown in Figure 8e‐f, the wound areas in the Cu/PMCS and Cu/PMCS+NIR groups were generally smaller than those in the other groups, especially on day 8. The wounds in the Cu/PMCS+NIR group were almost completely healed; however, none of the wounds in the other groups achieved complete healing. Quantitative analysis of the relative wound healing rates at different time points showed the fastest wound healing in the Cu/PMCS+NIR group, followed by the Cu/PMCS group (Figure 8g). HE staining (Figure 8h) also showed that the Cu/PMCS+NIR group formed a thicker and more complete epidermis, followed by the Cu/PMCS group, which was consistent with wound healing. Masson staining (Figure 8i) was used to characterize the formation of collagen fibers; the wound area in the Cu/PMCS+NIR and Cu/PMCS groups showed continuous and ordered collagen fibers, especially in the Cu/PMCS+NIR group. In contrast, in the other groups, the collagen fibers were more disorganized and sparse. Additionally, we selected CD68 expression as a marker of monocyte infiltration to detect inflammation in wounds. CD68 staining results showed that the Cu/PMCS+NIR and Cu/PMCS groups exhibited significantly reduced CD68 expression in the infected skin of mice wounds (red dots), with a relatively more significant reduction in the Cu/PMCS+NIR group (Figure 8j), which inhibited the inflammatory response at the wound site effectively. The quantitative analysis of CD68 is comparable to the above qualitative results (Figure S40a, Supporting Information). CD31 is an endothelial cell marker used to assess vascular regeneration in the wound area. A large number of CD31‐positive (red dots) cells appeared in the Cu/PMCS+NIR group, followed by the Cu/PMCS group; however, few CD31‐positive cells were found in the wound area of the other groups (Figure 8k), The above results are in line with the quantitative analysis of CD31 (Figure S40b), suggesting that Cu/PMCS promoted wound regeneration. In summary, Cu/PMCS effectively promoted the healing of infected wounds through the photothermal–Cu^2+^‐based synergistic antimicrobial effect.

## 3 Conclusion

In conclusion, a multifunctional Cu/PMCS nanoplatform was developed using a two‐step method, and the effect of Cu doping on PMCS was systematically investigated. According to the DFT theoretical calculations, Cu doping significantly improved the photothermal performance of the PMCS because of the introduction of impurity energy levels after doping with Cu ions, thus causing a new d‐orbital transition with strong spin‐orbit coupling. Additionally, the negative dielectric effect generated by transition‐induced LSPR enhanced the photothermal effect. Cu doping also improved the Fenton‐like catalytic activity and GSH depletion capacity of PMCS, which was beneficial for the CDT treatment. Notably, NIR irradiation further increased the catalytic activity and GSH depletion capacity of Cu/PMCS due to the increasing temperature and LSPR effect of Cu/PMCS. Finally, in vitro and in vivo biological experiments confirmed that Cu doping significantly improved the tumor‐suppressive and antibacterial abilities of PMCS and promoted wound healing. Therefore, Cu/PMCS is a multifunctional nanomaterial with antitumor, antibacterial, and wound‐healing properties, which can fully meet the needs of multiple clinical applications for melanoma treatment and repair and serve as a new idea for nanomaterials in the clinic.

## Conflict of Interest

The authors declare no conflict of interest.

## Author Contributions

L.L.: Conceptualization; Formal analysis; Data curation; Writing‐original draft. H.Z.: Data curation; Validation; Writing‐review & editing. S.X.: Data curation; Visualization. Y.Z.: Funding acquisition; Writing‐review & editing. Li Shangguan: Writing‐review & editing. C.W.: Methodology. F.P.: Conceptualization; Funding acquisition; Writing‐review & editing. X.L.: Conceptualization; Funding acquisition; Project administration.

## Supporting information

Supporting Information

## Data Availability

The data that support the findings of this study are available from the corresponding author upon reasonable request.
