# Supplementary material for: Copper‐Zinc Bimetallic Single‐Atom Catalysts with Localized Surface Plasmon Resonance‐Enhanced Photothermal Effect and Catalytic Activity for Melanoma Treatment and Wound‐Healing
Source: Adv Sci (Weinh). 2023 Apr 25;10(18):2207342. doi: 10.1002/advs.202207342 (PMC10288238; doi:10.1002/advs.202207342)
Supplement: Supplementary file 1 — Supporting Information [file ADVS-10-2207342-s001.pdf]

ADVANCED SCIENCE
Open Access

Supporting Information

for *Adv. Sci.*, DOI 10.1002/advs.202207342

Copper-Zinc Bimetallic Single-Atom Catalysts with Localized Surface Plasmon Resonance-Enhanced Photothermal Effect and Catalytic Activity for Melanoma Treatment and Wound-Healing

Lidan Liu, Haifeng Zhang, Shun Xing, Yu Zhang, Li Shangguan, Chao Wei, Feng Peng and Xuanyong Liu**

Copper-zinc bimetallic single-atom catalysts with localized surface plasmon resonance-enhanced photothermal effect and catalytic activity for melanoma treatment and wound-healing

Lidan Liu[a, b, #], Haifeng Zhang[a, d, #], Shun Xing[a, b], Yu Zhang[c], Li Shangguan[a, b], Chao Wei[a], Feng Peng[c, *], Xuanyong Liu[a, b, d, *]

[a]State Key Laboratory of High Performance Ceramics and Superfine Microstructure, Shanghai Institute of Ceramics, Chinese Academy of Sciences, Shanghai 200050, China

[b]Center of Materials Science and Optoelectronics Engineering, University of Chinese Academy of Sciences, Beijing 100049, China

[c]Medical Research Institute, Department of Orthopedics, Guangdong Provincial People's Hospital (Guangdong Academy of Medical Sciences), Southern Medical University, Guangzhou 510080, China

[d]School of Chemistry and Materials Science, Hangzhou Institute for Advanced Study, University of Chinese Academy of Sciences, 1 Sub-lane Xiangshan, Hangzhou 310024, China

# These authors contributed equally to this work

***Corresponding Author**
Xuanyong Liu, E-mail: xyliu@mail.sic.ac.cn
Feng Peng, Email: pengfeng@gdph.org.cn

1. Experimental section

1.1. Materials

Cu nitrate trihydrate ($Cu(NO_3)_2 \cdot 3H_2O$), Zn hexahydrate ($Zn(NO_3)_2 \cdot 6H_2O$), 2-methylimidazole and methanol were purchased from Aladdin Chemical (Shanghai, China). Ultrapure water was used in the experiments. All chemicals and solvents were of analytical grade and did not require further purification. All biological reagents were provided by Thermo Fisher Scientific.

1.2 Material synthesis

1.2.1 Synthesis of ZIF-Cu

Firstly, the zinc nitrate hexahydrate and copper nitrate trihydrate were weighed with different ratios (**Table S6**) and dissolved in 300 mL methanol, that was solution A. 13.14 g of 2-methylimidazole (160 mmol) was weighed and dissolved in 300 mL methanol, called solution B. Then solution B was poured into solution A and the mixed solution was stirred at room temperature for 3 h. After reaching the point, the mixed solution was centrifuged at 10000 rpm for 5 min to obtain the product ZIF-Cu, which was washed three times with methanol. Finally, the ZIF-Cu was freeze-dried for further use.

1.2.3 Synthesis of Cu/PMCS

Cu/PMCS was prepared by pyrolysis of ZIF-Cu, as follows: ZIF-Cu was first placed in a porcelain boat, and then the porcelain boat was placed in a horizontal tube furnace for heat treatment in an Ar atmosphere at 900 °C. The heating rate was 5 °C/min and the holding time was 120 min with the Ar flow of 3 sccm (cm^3/min). Undoped ZIF-8 after heat treatment was named PMCS, and different copper contents doped ZIF-8 after heat treatment (from low to high) were denoted as Cu/PMCS-1, Cu/PMCS-2, and Cu/PMCS-3. The samples were modified for photothermal testing and biological experiments by mixing with monomethoxy poly(ethylene oxide)-distearoyl phosphatidyl ethanolamine (mPEG-DSPE, 5 kD) in PBS solution for 24 h. Unless otherwise stated, Cu/PMCS in the context refers to Cu/PMCS-2.

1.3 Characterization

The morphology and size of PMCS and Cu/PMCS were characterized by scanning electron microscopy (SEM, Hitachi SU-8010), transmission electron microscopy (TEM, Hitachi 7700) and field emission TEM (JEM-2100F), respectively. X-ray powder

diffractometer (XRD, Bruker D8 Advance) and X-ray photoelectron spectrometer (XPS, ESCALAB MK II, with Mg Kα as excitation source) were used to characterize the phase structure, element existence state and metal loading of the samples. Using inductively coupled plasma optical emission spectrometry (ICP-OES) to measure metal ion release and quantify metal ions. Aberration-corrected HAADF-STEM images and energy dispersive spectroscopy (EDS) were obtained by spherical aberration electron microscopy (AC-HAADF-STEM). Nitrogen adsorption-desorption isotherms and corresponding pore size distributions were measured at 77 K using a Micromeritics ASAP 2460 system. The synchrotron radiation data were measured at the synchrotron radiation facility of the Shanghai Light Source. Raman spectroscopy measurements were performed at 514 nm excitation. The Zeta Potential/Particle System was used to measure hydrated particle size and zeta potential.

1.4 Material properties

1.4.1 Photothermal Property *in vitro*

Firstly, the 1 mL of PMCS, Cu/PMCS-1, Cu/PMCS-2, and Cu/PMCS-3 (100 μg/mL) dispersed in PBS were taken and placed in a 24-well plate, and then irradiated with 0.7 W/cm^2 under 808 nm NIR laser for 5 min, while the real-time temperature and thermal images were captured by a thermal imager camera. The *in vitro* photothermal properties of Cu/PMCS with different concentrations were investigated and the concentration range was 12.5, 25, 50, 100 μg/mL. Meanwhile, the heating curves of Cu/PMCS at different powers (0.5, 0.6, 0.7 and 0.8 W/cm^2) were also investigated.

1.4.2 Ion Quantification and Ion Release

The total amount of Zn and Cu ions in PMCS, Cu/PMCS-1, Cu/PMCS-2, and Cu/PMCS-3 was quantified by inductively coupled plasma atomic emission spectrometry (ICP-MS). Before tested, the material needs to be digested with aqua regia (V_{HCl}: V_{HNO3} = 3:1).

The release of Cu ions from Cu/PMCS in PBS solution at 37 °C was investigated by inductively coupled plasma atomic emission spectrometry (ICP-MS).

1.4.3 Determination of ·OH generation

The changes of absorbance of PMCS, Cu/PMCS-1, Cu/PMCS-2, and Cu/PMCS-3 (100 μg/mL) in PBS (pH = 6.5) were detected by the indicator agents TMB and hydrogen peroxide (H_2O_2)，which could reflect the ability to generate ·OH. At the same

time, the absorbance changes of Cu/PMCS in different pH (7.4 and 6.5) PBS and different concentrations of H_2O_2 (0.1, 1, 10 mM) were also detected.

The ability for Cu/PMCS to generate ·OH at different temperatures (37 ℃ and 56 ℃) and with and without NIR (808 nm) irradiation was also tested. Specifically, Cu/PMCS was mixed with H_2O_2 and TMB, the mixed solution was put into with 37 ℃ (noted as Cu/PMCS-37 ℃), 56 ℃ (noted as Cu/PMCS-56 ℃), or irradiated with NIR lase, where the mixed solution could be heated up to 56 ℃ (noted as Cu/PMCS-NIR (56 ℃). 5 min later, the changes of absorbance were detected.

1.4.4 Consumption of glutathione (GSH)

PMCS, Cu/PMCS-1, Cu/PMCS-2, and Cu/PMCS-3 (100 μg/mL) were mixed with GSH on a shaking table for 5 min under 37℃, and then 100 μL of the mixed solution was taken to react with the indicator agent DTNB. The changes in absorbance were measured with a microplate reader. In addition, the consumption of GSH by different concentrations of Cu/PMCS was also measured.

The ability for Cu/PMCS to consume GSH at different conditions (37 ℃, 56 ℃ and 808 nm NIR (56 ℃)) was also tested. Cu/PMCS was mixed with GSH (0.25, 0.5, 1 mM), and treated with different conditions for 5 min before tested.

1.5 DFT calculation

In this work, the first-principles calculation steps are completed through structural optimization, static self-consistent field (SCF), the density of state calculation, and charge calculation. All calculation steps are completed by VASP-6.1.0 [1], combined with the PBE functional[2, 3] under the generalized gradient approximation (GGA) [4] in the frame of density functional theory (DFT) with D3 dispersion correction [5], combined with the projector augmented wave (PAW) [6], and the plane wave cut off energy is 500 eV. Among them, the K point adopts the density of 9*9*1. In addition, for the calculation of the electromagnetic enhancement mechanism, we simulated the electromagnetic enhancement factor was simulated using the finite-difference time-domain (FDTD) method combined with the optical parameters calculated from the first-principles calculations.

1.6 Hemolysis test

The blood biocompatibility of PMCS and Cu/PMCS was tested using a hemolysis assay, and the blood was taken from 6-week-old female SD rat hearts and diluted with

PBS (V_{blood}:V_{PBS} = 4:5). The experimental steps are as follows: firstly, the materials were diluted to the corresponding concentration (100, 200, 400, 800 μg/mL) for use, and the negative and positive controls are PBS and deionized water, respectively. Secondly, placing 1mL of each group in a well plate (4 parallel wells), incubated at 37 °C for 30 min. Thirdly, 30 μL of diluted blood was added to it, and the incubation was continued for 1.5 h. And then 1 mL of the mixed solution was centrifuged at 3000 rpm for 5 min. Finally, 100 μL of supernatant was taken out to measure its absorbance (OD) at 545 nm, and the hemolysis rate (HR) was calculated according to the following formula:

$$\mathrm{HR} = \left(\frac{OD_{sample} - OD_{negative}}{OD_{positive} - OD_{negative}}\right) \times 100\%$$

1.7 *In vitro* cell experiments

The biocompatibility and anti-tumor properties of the materials were evaluated by cell proliferation and live-dead staining, and the *in vitro* biological effects of the material (100 μg/mL) were evaluated using L929, NIH3T3, HUVECs (normal cells) and B16F10 (tumor cells).

1.7.1 Cell culture

Mouse epidermal fibroblasts (L929), mouse embryonic fibroblasts (NIH3T3), human umbilical vein endothelial cells (HUVECs), and mouse skin melanoma cells (B16F10) were used to evaluate the *in vitro* biological effects of the materials. L929 and NIH3T3 medium consisted of basal medium (α-MEM, Gibco), 10% fetal bovine serum (FBS, Hyclone) and 1% penicillin/streptomycin (2A); HUVECs medium consisted of endothelial cell growth medium (ECM), 5.5% FBS, 1% 2A, and 1% cell growth factor (CGS, Hyclone); B16F10 medium consisted of basal medium (1640, Gibco), 10% FBS, and 1% 2A. During the culturing process, the cells were cultured in a 37 °C constant temperature cell incubator containing 5% CO_2, and passaged once every 2-3 days according to the cell state.

1.7.2 Biocompatibility

1.7.2.1 Cell proliferation

The Alamar Blue (AB) reagent was used to evaluate the effect of materials on cell proliferation. First, the cells were seeded in a 96-well transparent plate and cultured in an incubator for 24 h with a seeding density of 1×10^4 cells/well. Then, the original medium was replaced with a fresh medium containing the material (100 μg/mL). After

the material was incubated with the cells for 24 h, the cells were incubated with 10% AB for 2 h. Finally, the fluorescence intensity value of each well was measured by a microplate reader, and the test wavelength was 560 nm (Ex) / 590 nm (Em). The corresponding cell inhibition rate was calculated according to the following formula:

$$\text{Cell ability} = \left(\frac{F - F_{blank}}{F_0 - F_{blank}}\right) \times 100\%$$

F represented the fluorescence intensity of the medium after the cells are co-cultured with the material;

F_0 indicated the fluorescence intensity of the medium that the cells are not co-cultured with the material;

F_{blank} represented the fluorescence intensity of the medium containing only 10% alamar blue.

1.7.2.2 Cell live/dead staining

The cells were fluorescently stained using a live/dead cell staining kit (Thermo Fisher Scientific Inc., USA). Firstly, the cells were seeded in a 24-well plate for 24 hours with a seeding density of 5×10^4 cells/well. Then, the original medium was replaced with fresh medium containing material (100 μg/mL) and the culture was continued for 24 h. Finally, the diluted dye was used for staining, and the fluorescence microscope was used to observe and record.

1.7.3 *In vitro* antitumor properties

1.7.3.1 Cell proliferation

The procedure of cell proliferation experiment of tumor cells (B16F10) is similar to that of normal cells, please refer to 1.7.2.1. However, for the photothermal group, photothermal treatment (808 nm NIR, 0.7 W/cm^2, 5 min) was required after adding materials, and the effect of different laser powers on the viability of B16F10 cells was also tested.

1.7.3.2 Cell live/dead staining

The live and dead staining procedure of tumor cells (B16F10) is similar to that of normal cells, please refer to 1.7.2.2. But the photothermal group needs photothermal treatment after adding the material.

1.7.3.3 Flow Cytometric Analysis of Apoptosis

First, B16F10 cells were inoculated in a 24-well plate for 24 h, and the inoculation density was 5×10^4 cells/well. Then, it was changed to fresh medium containing material (100 μg/mL), treated with light and no light. After continuing the culture for 24 h, the

cells were digested with trypsin, different parallel samples of cells were collected together, then the cell precipitate was collected by centrifugation and washed several times. After that, the cells and nuclei were stained with Annexin V-FITC apoptosis detection kit and propidium iodide respectively, and finally the results of apoptosis and cell cycle were detected and analyzed by flow cytometry.

1.7.3.4 Intracellular GSH assay

B16F10 cells were seeded in a 24-well plate, and the material was added after 24 h of incubation, treated with or without light. Incubated for 24 h, the GSH detection kit (Biyuntian) was used to measure the level of intracellular GSH.

1.7.3.5 Intracellular ROS staining

B16F10 cells were seeded in a 24-well plate for 24 h, and the material was added, treated with or without light. After 6 h, the DCFH-DA stain was used to measure the level of ROS in B16F10 cells and observe the cells by fluorescence microscope.

1.7.4 Cell Migration

Cell migration was assessed by scratch assay, and nuclei were stained with 4',6-diamidino-2-phenylindole (DAPI, Invitrogen, USA), observed by fluorescence microscopy. Calculate the cell migration rate according to the formula shown below:

$$\text{Cell migration} = \left(\frac{A_0 - A_t}{A_0}\right) \times 100\%$$

A_0 represents the scratched area at 0 h;

A_t is the scratched area without cell migration at different time points;

1.7.5 Endothelial tubule formation assay

The effect of Cu/PMCS on the angiogenic properties of HUVECs was evaluated by tubulogenesis experiments. Matrigel basement membrane matrix (BD, Biosciences) and brightfield strips of fluorescence microscopy were used to observe microtubule formation.

1.7.6 Real-time quantitative PCR (RT-qPCR)

The effect of Cu/PMCS on the expression of angiogenesis and tissue healing-related genes (*VEGFA*, *TGFB1*, *Colla1* and *FGF2*) in L929 and *VEGF* in HUVECs were further evaluated using RT-qPCR. The experimental steps were as follows: the cells were seeded in a 6-well plate with a density of 1×10^5 cells per well for 24 h, and the medium were replaced with fresh medium containing materials for another 24 h. After washed twice with PBS, total RNA was extracted according to the Trizol (SigmaAldrich, USA) method and quantified by nanodrop 2000 (Thermofisher, USA).

Then 1 μg of total RNA was reverse transcribed into cDNA using the st Strand cDNA synthesis supermix Kit (Yeasen, China). The synthesized cDNA was then mixed with SYBR Green Mastermix and primers to quantify gene expression of the target gene. Gene analysis used GAPDH as the reference gene and normalization and the $2^{-\Delta\Delta CT}$ method was used to relative quantification. Primer sequences are listed in **Table S7-8**.

1.8 *In vitro* bacterial experiments

1.8.1 Bacterial inoculation

The material and bacterial solution were diluted to the corresponding concentration with PBS, obtaining 4 mL of the mixed bacterial solution in total. The final concentration of the material was 100 μg/mL. Bacterial species include Gram-positive Staphylococcus aureus (*S. aureus*) and Gram-negative Escherichia coli (*E. coli*).

1.8.2 Photothermal antibacterial

The photothermal antibacterial properties of PMCS and Cu/PMCS (100 μg/mL) were evaluated by bacterial colonies counting, bacterial live-dead staining, and bacterial morphological observation. The photothermal treatment was irradiated with 808 nm NIR laser for 5 min (0.7 W/cm^2).

1.8.2.1 Bacteria colonies counting

Firstly, 1 mL of the mixed bacterial solution was taken out and placed in a 24-well plate, performing photothermal and non-photothermal treatment. After sucking out and diluting the bacterial liquid with NaCl to a suitable concentration, 100 μL of the diluted bacteria was taken out with a pipette gun and placed on the agar plate, spread evenly by a push rod. Then, the agar plates were placed in a 37 ℃ incubator for about 18 h. Finally, take a picture for record and count analysis.

1.8.2.2 Bacterial live/dead staining

Bacteria were fluorescently stained using a bacterial live/dead staining kit (L13152, Molecular Probes, USA). First, the bacteria were inoculated in a 24-well plate with glass slides, cultured in a 37 °C incubator. After the bacteria adhere and spread on the glass slides, 1 mL of material (100 μg/mL) was added under light or dark. Next, the bacteria were fluorescently stained with the diluted bacterial live/dead staining reagent and observed with a fluorescence microscope.

1.8.2.3 Observation of bacterial morphology

The previous steps were the same as the bacteria live/dead, but the bacteria were

fixed with 2.5% glutaraldehyde after light treatment. After 4 h of fixation, the bacteria were dehydrated with different concentrations of alcohol. Finally, the morphology of bacteria was observed by scanning electron microscope.

1.8.3 Physical antibacterial

The physical antibacterial properties of different materials (100 μg/mL) were evaluated by bacterial plating, and the inoculation density of *S. aureus* and *E. coli* was 10^5 CFU/mL. Please refer to 1.8.2.1 for detailed steps of bacteria colonies counting.

1.9 Animal experiments

All experimental procedures performed on animals were in accordance with the guidelines of the Animal Research Committee of Guangdong Provincial People's Hospital (number: KY-D-2021-272-02).

1.9.1 Evaluation of tumor-inhibiting ability *in vivo*

The antitumor activity of Cu/PMCS was evaluated using the B16F10 tumor-bearing mouse model. Briefly, mice with tumor volumes around 50 mm^3 were randomized into 6 groups (n = 4) for treatment, grouped as follows: 1) Control, 2) Control+NIR, 3) PMCS, 4) PMCS+NIR, 5) Cu/PMCS and 6) Cu/PMCS+NIR, the NIR group was irradiated with 808 nm laser (0.7 W/cm^2) for 5 min, when the tumor surface temperature changes and thermal images were recorded by a photothermal camera (Fluke Ti450). Mice received intratumoral injections of equal amounts (2 mg/kg) of each material every two days, but were only photothermally treated on Day 0 and Day 2. Tumor volumes and mouse body weights were measured every two days, and relative tumor volumes were calculated too. When the tumor volume of mice in the control group exceeded ethically, all mice were sacrificed, and the tumor site and major organs such as tumor, heart, spleen, lung and kidney were sectioned and stained.

1.9.2 Evaluation of antibacterial and wound healing ability *in vivo*

The *in vivo* antibacterial and wound healing abilities of Cu/PMCS were evaluated using the S. aureus infection model in BALB/c female mice. Briefly, 20 μL of *S. aureus* solution (10^8 CFU/mL) was introduced into a circular wound (8 mm × 8 mm) on the back of mice to establish an infection model. After 6 h of infection, mice were randomized into 6 groups (n = 4) for treatment, grouped as follows: 1) Control, 2) Control+NIR, 3) PMCS, 4) PMCS+NIR, 5) Cu/PMCS and 6) Cu/PMCS+NIR. After the treatment, wounds were measured and photographed every two days. Finally, the mice were sacrificed at the corresponding time points, and the wound skin tissue was

taken out for plating and section staining. The wound healing rate was calculated as follows:

$$\% \text{ Wound area closed} = \left\{ 1 - \frac{\left(\frac{\text{wound area}}{\text{splint area}}\right) \text{Day x}}{\left(\frac{\text{wound area}}{\text{splint area}}\right) \text{Day 0}} \right\} \times 100$$

1.10 Statistic analysis

All data were presented as mean ± standard deviation (SD). Statistical analysis was performed using two-way ANOVA, one-way ANOVA and t-test followed by Tukey's post hoc test via SPSS 19.0 software. $^{*}p < 0.05$; $^{**}p < 0.01$; $^{***}p < 0.001$.

2. Supplementary Figure

As shown in **Fig. S1**, the XRD patterns (**Fig. S1a**) and TEM images (**Fig. S1b**) are consistent with literature, confirming the successful preparation of Cu/ZIF [7, 8].

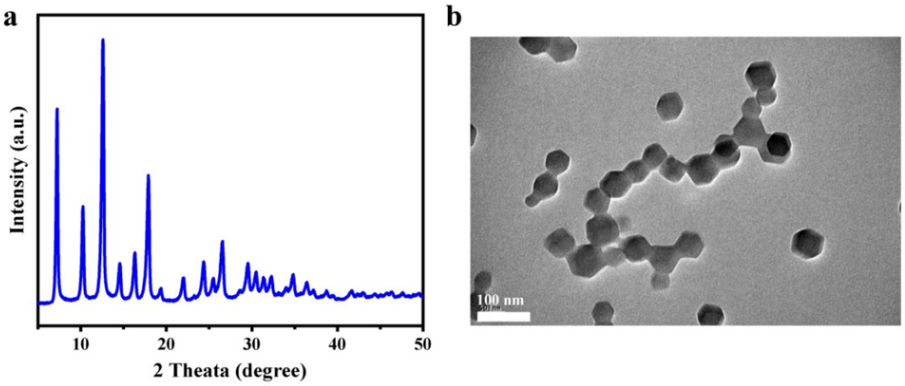

Fig. S1. XRD patterns (a) and TEM image (b) of ZIF-Cu.

The **Fig. S2** showed that the hydrated particle size of Cu/PMCS was 68.1 nm, and PDI was 0.340. PDI lower than 0.5 means good dispersion.

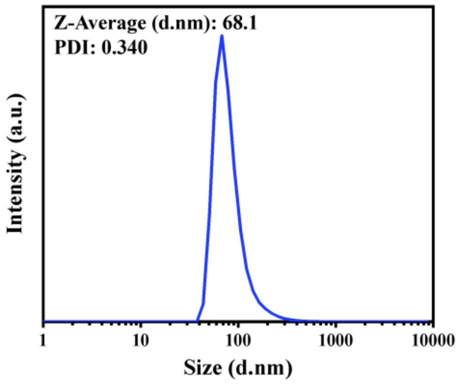

Fig. S2. Particle size distribution of Cu/PMCS.

The zeta potential of PEGylated Cu/PMCS (**Fig. S3**) changed from -15 to -10 mV, which demonstrated the successful modification of mPEG-DSPE [7].

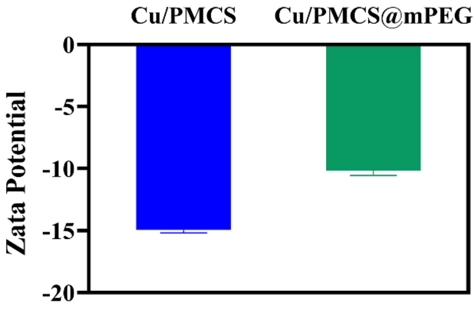

Fig. S3. The zeta potential of Cu/PMCS and Cu/PMCS@mPEG.

The AC-HAADF-STEM image of PMCS showed the absence of clusters and particles in PMCS and clearly visible individual atoms (bright white dots, highlighted by red circles) loaded on N-doped carbon matrix carriers, which could be single Zn atom (**Fig. S4**).

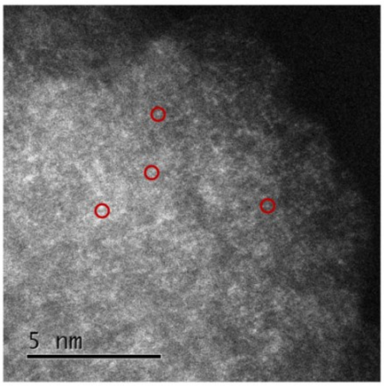

Fig. S4. AC-HAADF-STEM image of PMCS.

The results of synchrotron radiation showed that the Zn in both PMCS and Cu/PMCS had a single atomic structure (**Fig. S5**).

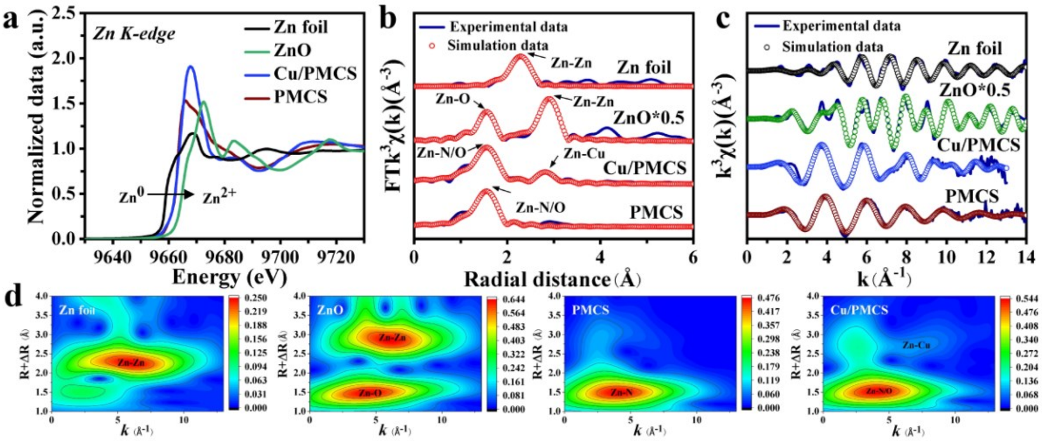

Fig. S5. (a) XANES spectra; (b-c) Fourier transformations in R-space (b) and k-space (c); (d) Wavelet transform (WT) of Zn foil, ZnO, PMCS and Cu/PMCS.

As shown in **Fig. S6**, the XRD patterns (**Fig. S6a**) and TEM images (**Fig. S6b**) confirmed the successful preparation of Cu/ZIF with different contents of copper. Additionally, no significant changes in the crystallinity and morphology of ZIF were found after copper doping.

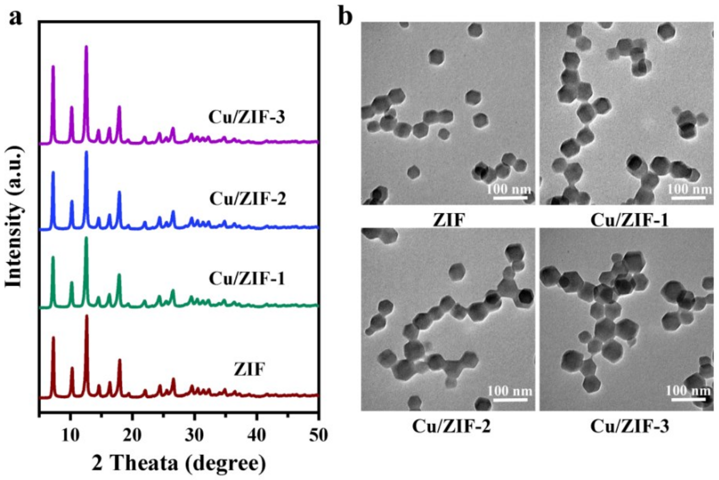

Fig. S6. XRD patterns (a) and TEM image (b) of ZIF, Cu/ZIF-1, Cu/ZIF-2 and Cu/ZIF-3.

The XRD patterns (**Fig. S7a**) and TEM images (**Fig. S7b**) proved the successful preparation of PMCS, Cu/PMCS-1, Cu/PMCS-2 and Cu/PMCS-3. In addition, there are no significant changes among them. The XPS full spectra (**Fig. S7c**) showed that they were mainly composed of carbon, nitrogen and oxygen. And Cu 2p XPS spectra (**Fig. S7d**) semi-quantitative results showed that the copper content increased sequentially from PMCS, Cu/PMCS-1, Cu/PMCS-2 to Cu/PMCS-3.

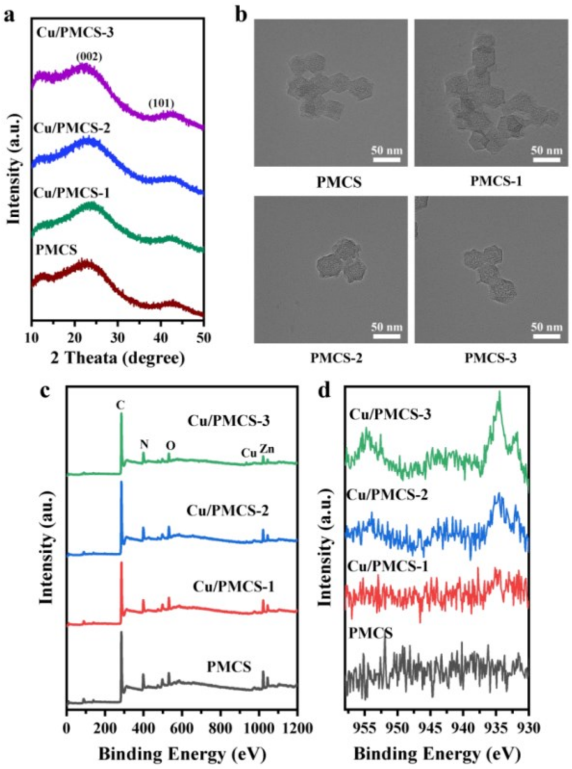

Fig. S7. XRD patterns (a), TEM image (b), XPS full spectra (c) and Cu 2p XPS spectra (d) of PMCS, Cu/PMCS-1, Cu/PMCS-2, Cu/PMCS-3.

The **Fig. S8** shows that the metals are in the single atom state in PMCS, Cu/PMCS-1 Cu/PMCS-2 and Cu/PMCS-3.

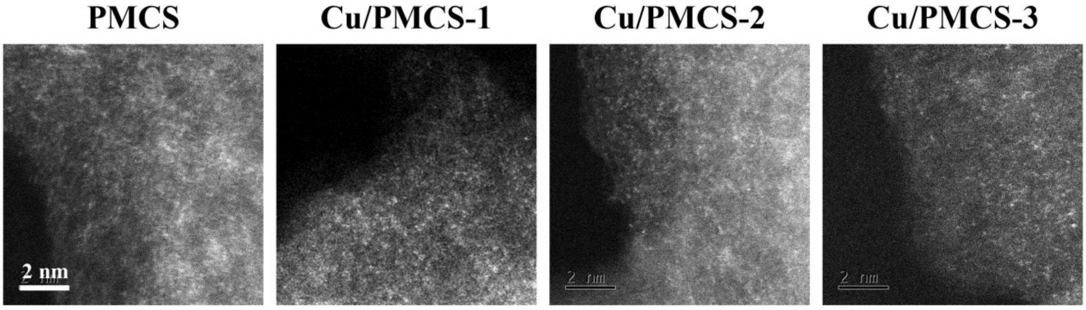

Fig. S8. High-angle annular dark-field scanning transmission electronmicroscopy with aberration-corrected (ac-HAADF-STEM) images of PMCS, Cu/PMCS-1, Cu/PMCS-2, and Cu/PMCS-3 (isolated bright dots may be pairs of Zn and Cu single-atoms).

Fig. S9 showed the infrared thermal images corresponding to the heating curve of PMCS, Cu/PMCS-1, Cu/PMCS-2, Cu/PMCS-3 *in vitro* (**Fig. 2b**).

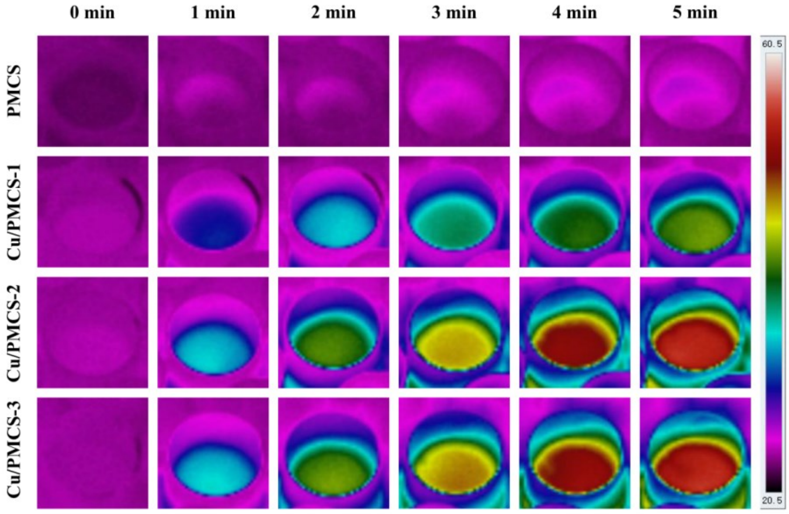

Fig. S9. Infrared thermographic images corresponding to the heating curves of PMCS, Cu/PMCS-1, Cu/PMCS-2, and Cu/PMCS-3.

Plot of cooling time versus negative natural logarithm of the temperature driving force obtained from the cooling stage is shown in **Fig. S10**, which gave time constants (τ_s) of 253.4, 242.31, 251.91, and 233.66 for PMCS, Cu/PMCS-1, Cu/PMCS-2, and Cu/PMCS, respectively.

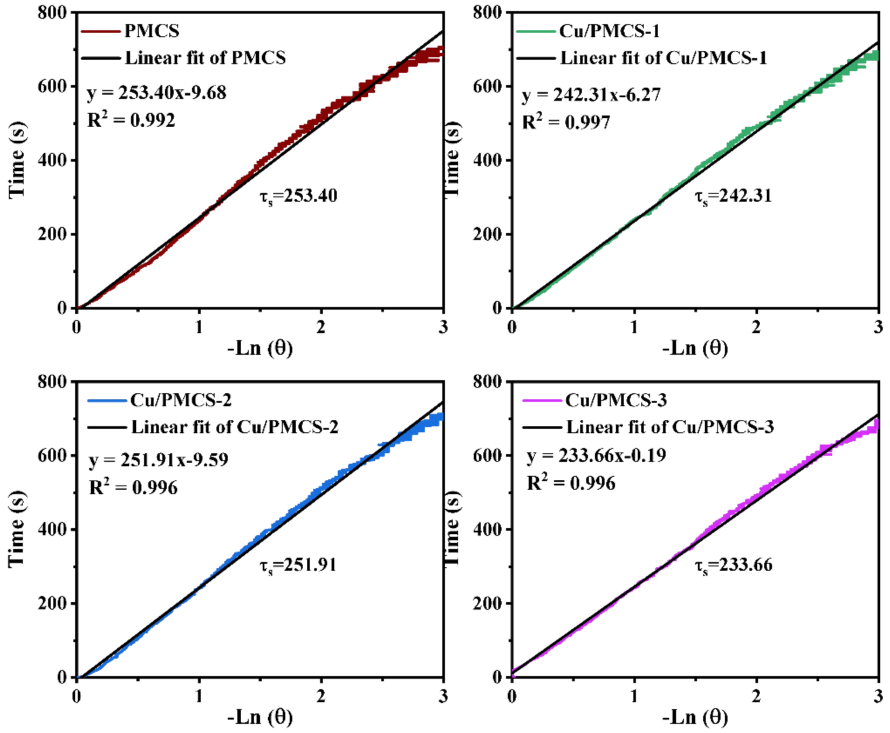

Fig. S10. Plot of cooling time versus negative natural logarithm of the temperature driving force obtained from the cooling stage of PMCS, Cu/PMCS-1, Cu/PMCS-2, Cu/PMCS-3.

As shown in **Fig. S11**, the higher the concentration, the faster the temperature increased, indicating that the temperature increase of Cu/PMCS was concentration-dependent.

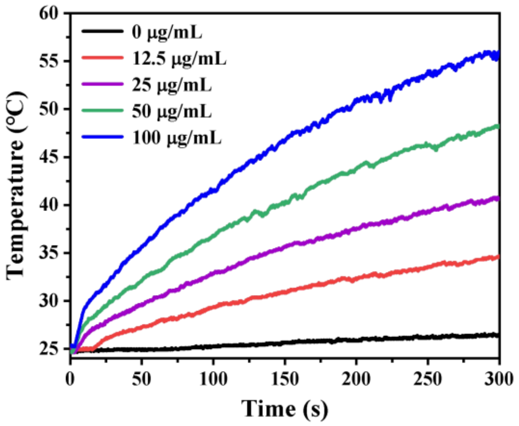

Fig. S11. Heating curves of Cu/PMCS with different concentrations.

The temperature increase of Cu/PMCS could be controlled by adjusting the power of laser (**Fig. S12**).

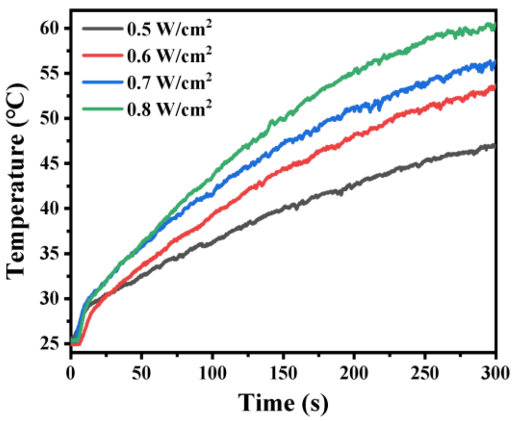

Fig. S12. Heating curves of Cu/PMCS at different laser powers.

Fig. S13 showed the heating-cooling curves of Cu/PMCS for 5 cycles *in vitro*, indicating that Cu/PMCS had good photothermal stability.

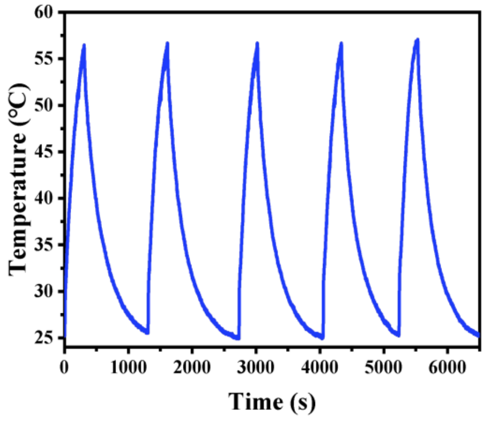

Fig. S13. Heating-cooling curves of Cu/PMCS for 5 cycles.

As shown in **Fig. S14**, the photothermal properties were enhanced with increasing temperature, but 900 °C was the best, which was consistent with the previous report.

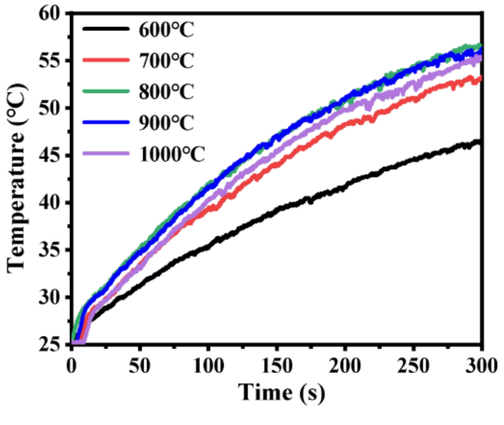

Fig. S14. Heating curves of Cu/PMCS heat-treated at different carbonization under 808 nm NIR irradiation with power density of 0.7 W/cm^2

As shown in **Fig. S15**, the bluer the color of the solution, the more ·OH was produced, which indicated that the higher the copper concentration of the material, the stronger the ability to produce ·OH.

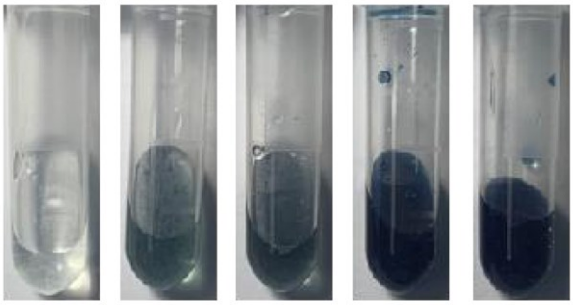

Fig. S15. Photos of the solution tested by TMB corresponding to Fig. 3b.

The catalytic activity of Cu/PMCS at different concentrations was tested. The results of **Fig. S16** showed that the higher the concentration of Cu/PMCS, the stronger the ability to produce ·OH.

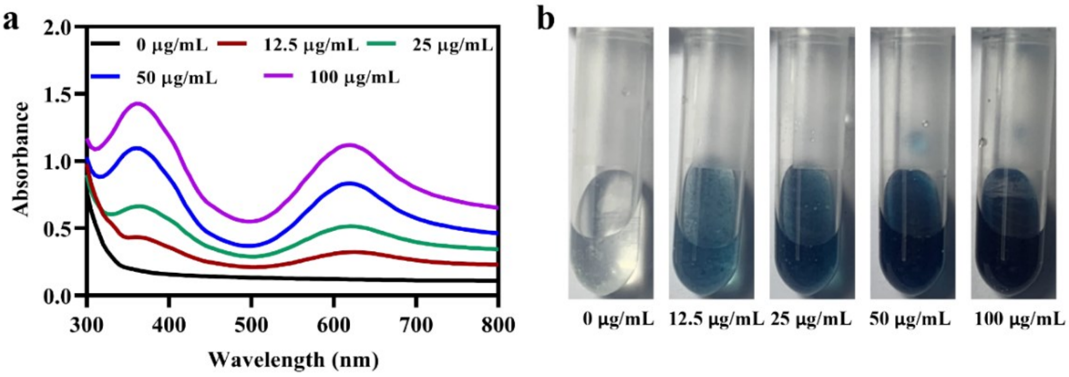

Fig. S16. The ability to produce ·OH of Cu/PMCS with different concentration tested by TMB: absorption spectrum (a), and photos of the solution (b).

The catalytic activity of Cu/PMCS at different pH values was tested, F**ig. S17** showed that the catalytic efficiency of Cu/PMCS-2 was significantly higher under acidic conditions.

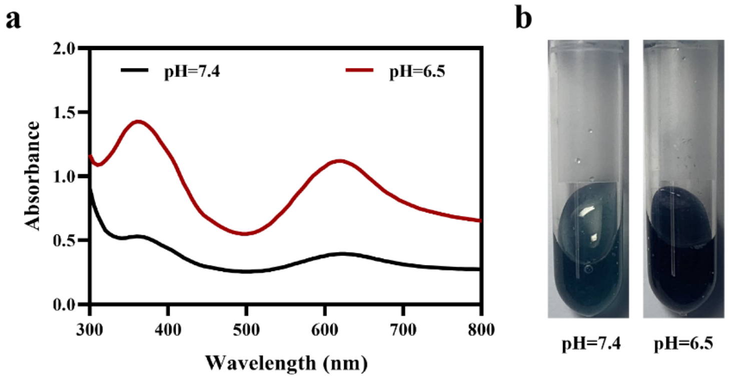

Fig. S17. The ability to produce ·OH of Cu/PMCS at different pH: absorption Spectrum (a), and photos of the solution (b).

The catalytic activity of Cu/PMCS at different concentrations of hydrogen peroxide was tested. As shown in **Fig. S18**, the higher the concentration of H_2O_2, the stronger the ability for Cu/PMCS to produce ·OH.

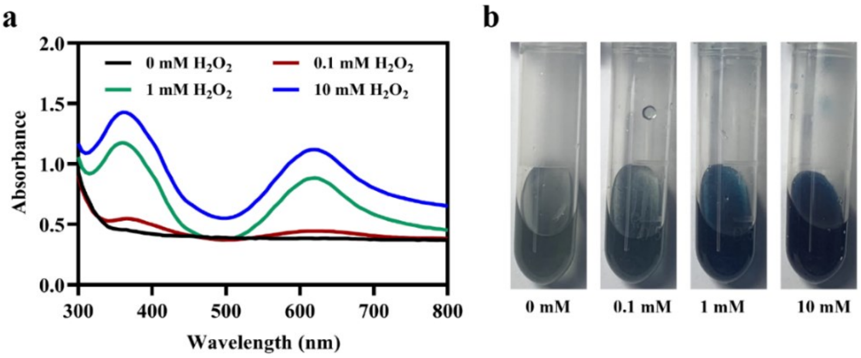

Fig. S18. The ability to produce ·OH of Cu/PMCS at different concentration of H_2O_2: absorption Spectrum (a), and photos of the solution (b).

As shown in **Fig. S19**, the more yellow the color of the solution, the less GSH is consumed, which indicates that a higher copper content of the material have the greater ability to consume GSH.

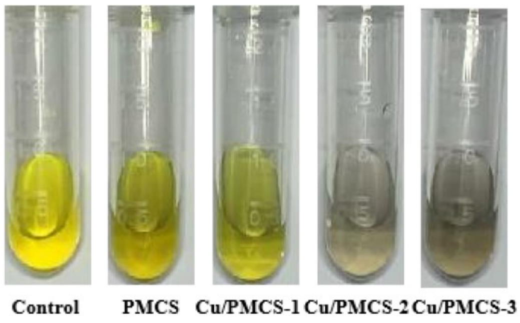

Fig. S19. Photos of the solution tested by DTNB corresponding to Fig. 3c.

The GSH depletion ability of Cu/PMCS at different concentrations was tested. The results of **Fig. S20** showed that the higher the concentration of Cu/PMCS, the stronger the ability to consume GSH.

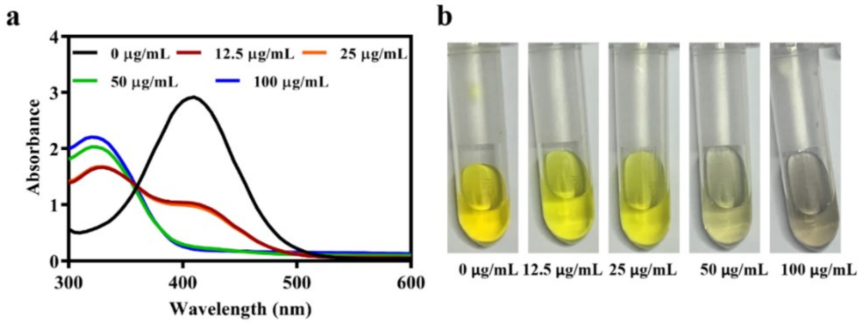

Fig. S20. The ability to consume GSH of Cu/PMCS with different concentration tested by DTNB: absorption Spectrum (a), and photos of the solution (b).

Fig. S21 showed the pore size of PMCS, Cu/PMCS-1, Cu/PMCS-2, and Cu/PMCS-3, indicating that with the increase of Cu content, the pore size became smaller significantly.

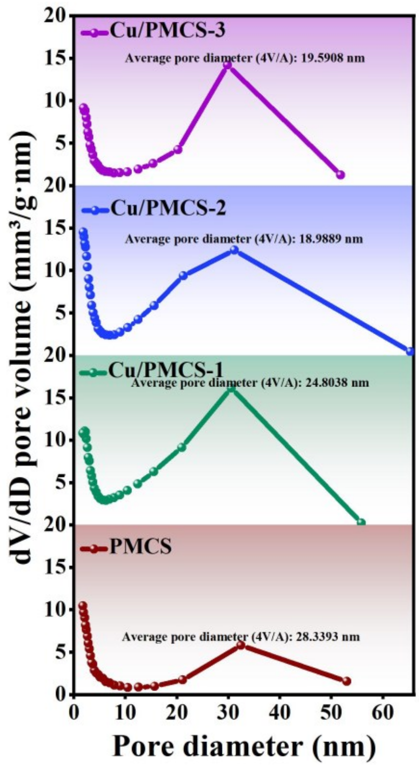

Fig. S21. The pore size distribution of PMCS, Cu/PMCS-1, Cu/PMCS-2 and Cu/PMCS-3 test by BET.

The results showed that at a low hydrogen peroxide concentration (0.1 mM), the peroxidase activity was significantly higher at 56 ℃ than at 37 ℃, indicating that elevated temperature increased the peroxidase activity of Cu/PMCS (**Fig. S22a** and **Fig. S22c**), However, there was no significant difference in the peroxidase activity of Cu/PMCS at a high hydrogen peroxide concentration (0.1 mM) (**Fig. S22b** and **Fig. S22d**), which may be due to the different catalytic rates at different hydrogen peroxide concentrations. It is noteworthy that NIR light irradiation further enhanced the peroxidase activity of Cu/PMCS at both low and high hydrogen peroxide concentrations.

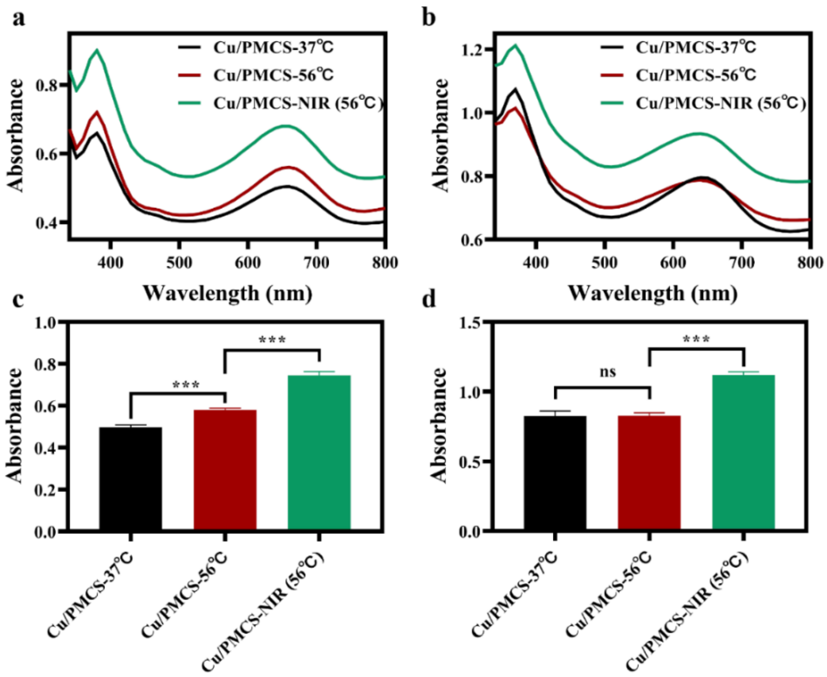

Fig. S22. Qualitative (a, b) and Quantitative analysis (c, d) of the produced ·OH by the Cu/PMCS-37℃, Cu/PMCS-56℃ and Cu/PMCS-NIR (56℃) groups at different concentration of H_2O_2: 0.1 mM (a, c) and 1mM (b, d). Data represent means±SD (n = 3). Statistical significance was calculated by one-way ANOVA analysis. *$p < 0.05$; **$p < 0.01$; ***$p < 0.001$.

The results showed that increasing the temperature enhanced the GSH depletion capacity of Cu/PMCS at different GSH concentrations. Similarly, the GSH depletion capacity of Cu/PMCS was further enhanced under NIR light irradiation (**Fig. S23**).

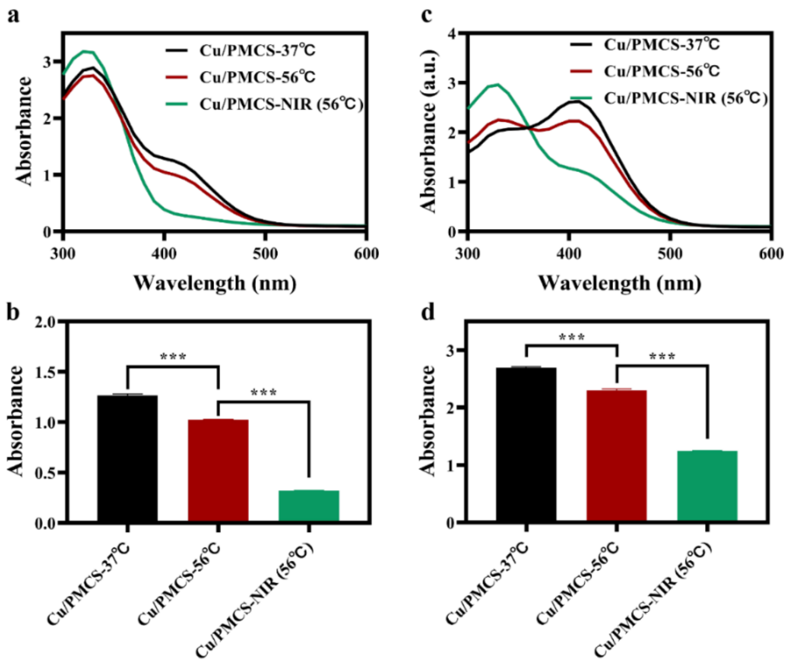

Fig. S23. Qualitative (a, b) and Quantitative analysis (c, d) of the ability to consume GSH of Cu/PMCS-37℃, Cu/PMCS-56℃ and Cu/PMCS-NIR (56℃) at different concentration of GSH: 0.5 mM (a, c) and 1mM (b, d). Data represent means ± SD (n = 4). Statistical significance was calculated by two-way ANOVA analysis. *p < 0.05; **p < 0.01; ***p < 0.001.

The TMB reagent was used to detect the production of ROS. The higher the absorbance of TMB, the more ROS are produced. As shown in **Fig. S24a**, the ROS generation also increased gradually with the increase of temperature, among which 800 ℃ and 900 ℃ were relatively better. In addition, the DTNB reagent was used to detect the depletion of GSH. The lower the absorbance of DTNB at around 412 nm, the higher the depletion of GSH. **Fig. S24b** showed that GSH is consumed more with increasing temperature, where 800 °C, 900 °C and 1000 °C are almost the same. In summary, 900°C was chosed as the heat treatment temperature.

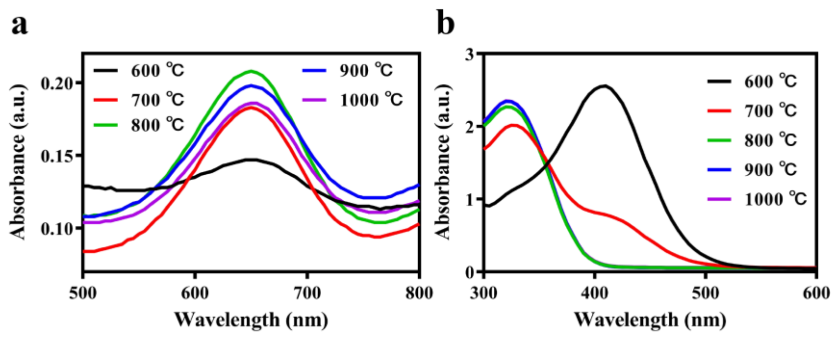

Fig. S24. The absorbance spectra of TMB for detecting the ability of Cu/PMCS heat-treated at different carbonization temperatures to produce ·OH, and (c) The absorbance spectra of DTNB for detecting GSH consumption of Cu/PMCS heat-treated at different carbonization temperatures.

As shown in **Fig. S25**, a higher irradiation power caused lower the cell viability, indicating a better inhibitory effect. The cell viability was approximately 53% when the power was 0.7 W/cm^2, and 0.7 W/cm^2 was the power used for subsequent experiments.

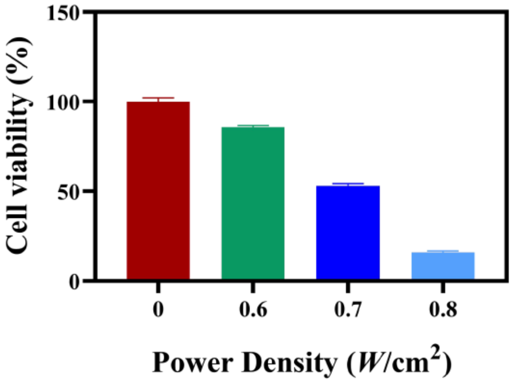

Fig. S25. Effects of Cu/PMCS at different laser powers on the viability of B16F10 cells. Data represent means ± SD (n = 4).

The cell cycle results (**Fig. S26**) showed that the percentage of cells in G2 phase was different in different groups, in which Cu-BTC@PDA was the lowest, indicating that CDT/PTT mainly blocked cell growth in the S-phase.

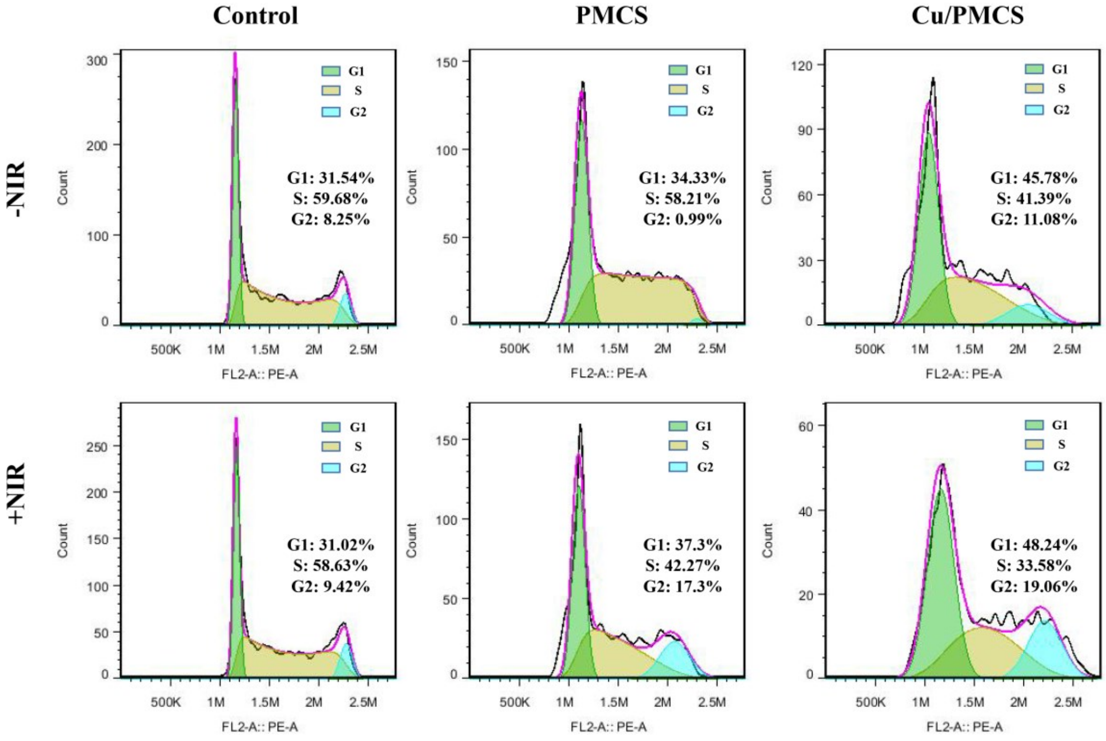

Fig. S26. Flow cycle diagram of B16F10 cells.

Fig. S27 was an infrared thermal image corresponding to the heating curve of mouse in tumor model (**Fig. 5b**).

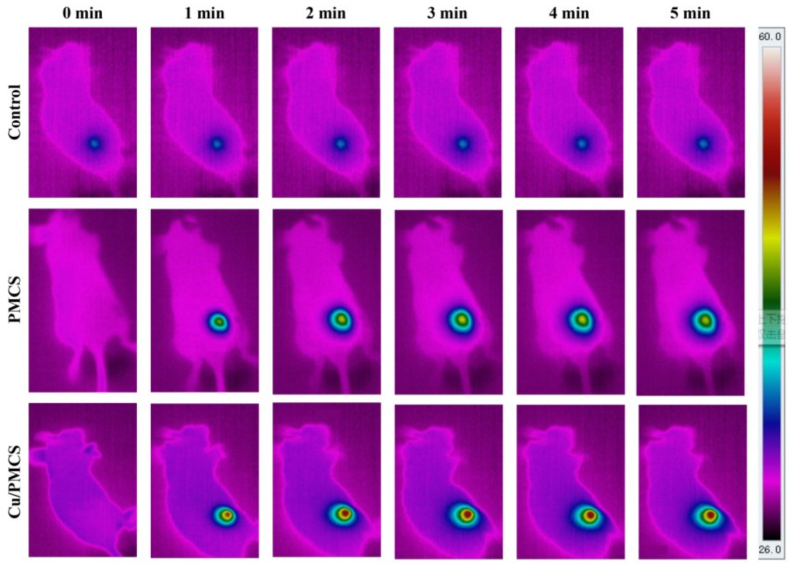

Fig. S27. Infrared thermographic images in tumor model.

It could be clearly seen in **Fig. S28** that the Cu/PMCS group had a significant inhibitory effect on melanoma compared to the PMCS group, which was the effect of CDT treatment. More importantly, Cu/PMCS almost completely inhibited melanoma growth in the presence of NIR, indicating that Cu/PMCS had excellent CDT/PTT synergistic anti-melanoma effect.

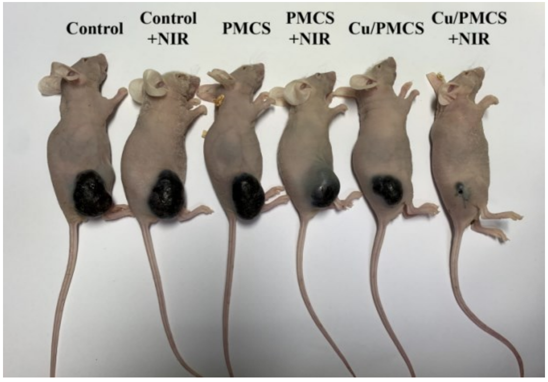

Fig. S28. Photos of mice treated for 15 days

The green signal in TUNEL images represented apoptosis cells, so the green signal was quantified to the meaning of the TUNEL staining clearly. **Fig. S29** indicated that the Cu/PMCS group showed partial apoptosis, compare with the Control and PMCS group, while the Cu/PMCS+NIR group exhibited more extensive apoptosis, which is consistent with qualitative results (**Fig. 5g**).

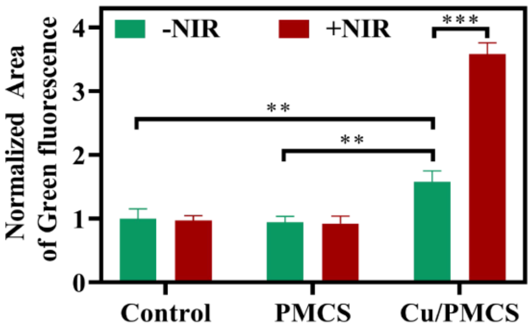

Fig. S29. Quantitative analysis of TUNEL staining. Data represent means ± SD (n = 3). Statistical significance was calculated by two-way ANOVA analysis. *p < 0.05; **p < 0.01; ***p < 0.001.

There were no signs of toxicity in the histological analyses of major organ sections, including the heart, liver, spleen, lung, and kidney in H&E staining pictures (**Fig. S30**), indicating that Cu/PMCS had good *in vivo* biocompatibility.

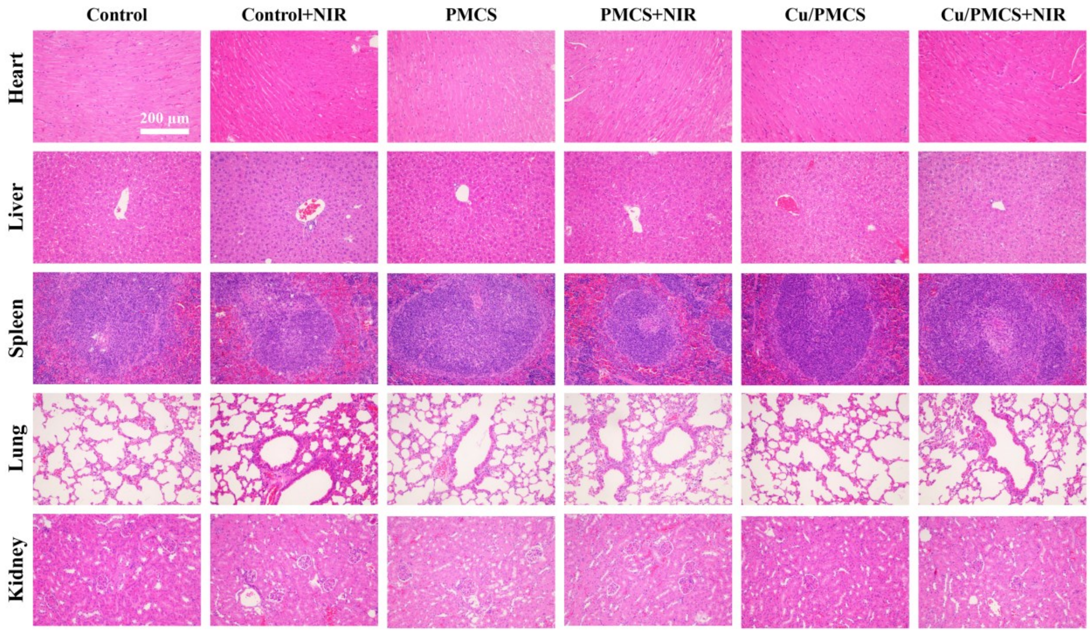

Fig. S30. H&E staining pictures of major organs in tumor-bearing mice.

Furthermore, we tested the sustained antimicrobial effects of PMCS and Cu/PMCS. Specifically, the bacteria were co-cultured with the material for 24 h, followed by bacterial colony counting and live-dead staining tests, and the results are shown in **Fig. S31**. Compared to PMCS, Cu/PMCS had a significant, sustained antimicrobial effect attributed to the release of Cu^{2+} from Cu/PMCS (**Table. S6**).

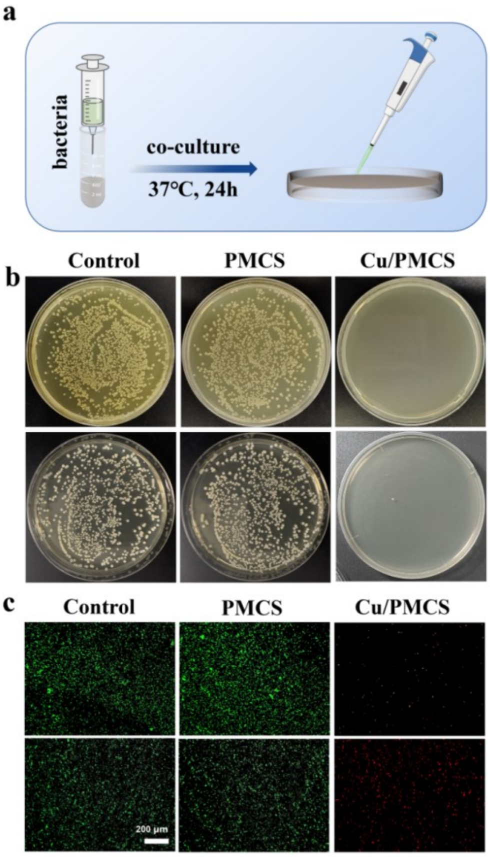

Fig. S31. Images of *E. coli* and *S. aureus* colonies. Live-dead staining of (j) *E. coli* and (k) *S. aureus* to determine the persistent antibacterial effect.

To further investigate the effect of copper ion release on the photothermal performance and catalytic activity of Cu/PMCS, the performance of Cu/PMCS was compared with that of Cu/PMCS soaked in PBS for 24 h (noted as Cu/PMCS-24h). It was shown that there was no significant change in the photothermal performance, ·OH production and GSH consumption of Cu/PMCS and Cu/PMCS-24h, indicating that the effect of copper ion release on the photothermal performance and catalytic activity of Cu/PMCS was minimal (**Fig. S32**). This is due to the small amount of copper ions released and the fact that there is almost no significant increase in the amount of copper ions released with increasing time (**Table. S5**).

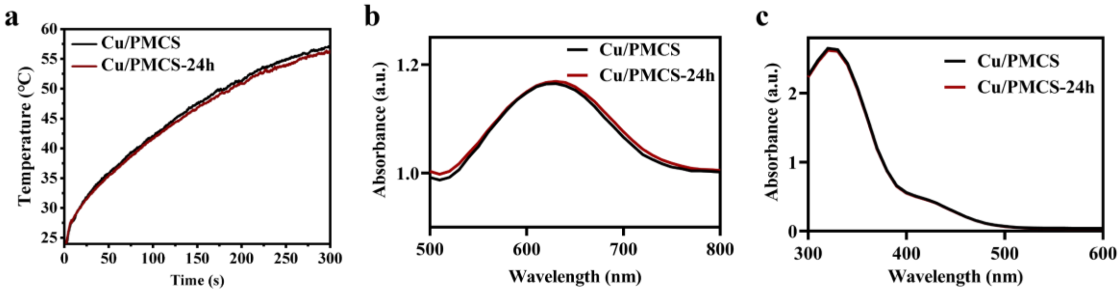

Fig. S32. (a) Heating curves of Cu/PMCS and Cu/PMCS-24h under 808nm NIR irradiation with power density of 0.7 W/cm^2, (b) The absorbance spectra of TMB for detecting the ability of Cu/PMCS and Cu/PMCS-24h to produce ·OH, and (c) The absorbance spectra of DTNB for detecting GSH consumption of Cu/PMCS and Cu/PMCS-24h. (Cu/PMCS soaked in PBS for 24 h was noted as Cu/PMCS-24h)

The antitumor effect of Cu/PMCS under bacterial-tumor coexistence *in vitro* was evaluated by co-culturing mouse melanoma cells (B16f10) with bacteria (*E. coli* and *S. aureus*). The proliferation results (**Fig. S33**) showed that both Cu/PMCS and Cu/PMCS+NIR groups had significant inhibitory effects on tumor cells and bacteria compared with other groups, and Cu/PMCS+NIR had more than 90% inhibition of tumor cells and bacteria, which was consistent with the results of cells and bacteria cultured separately.

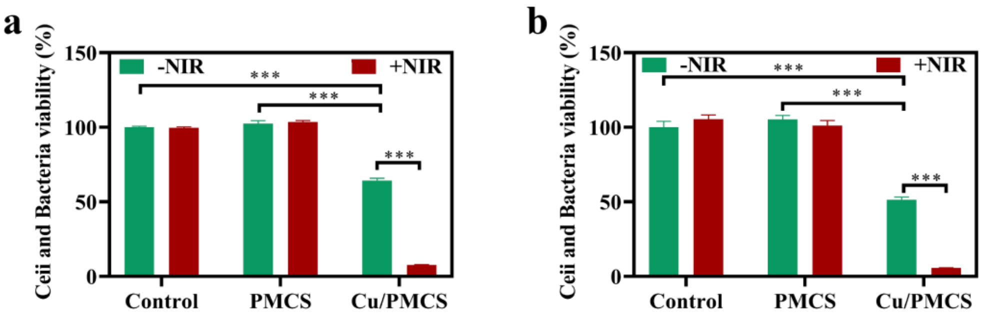

Fig. S33. The Viability of cells co-cultured with *E. coli* (a) and *S. aureus* (b). Data represent means ± SD (n = 4). Statistical significance was calculated by two-way ANOVA analysis. *p < 0.05; **p < 0.01; ***p < 0.001.

The NIH3T3 cell proliferation and the live-dead cell staining results also showed that Cu/PMCS had excellent biocompatibility (**Fig. S34**).

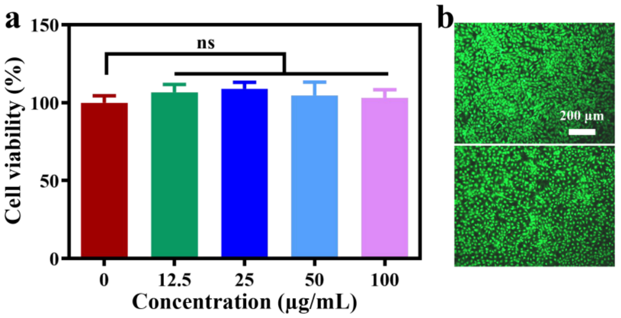

Fig. S34. Cytotoxicity of Cu/PMCS on NIH3T3 cells: (a) Cell Proliferation and (b) live-dead staining. Data represent means ± SD (n = 4). Statistical significance was calculated by one-way ANOVA analysis. *p < 0.05; **p < 0.01; ***p < 0.001.

Fig. S35 is the hemolysis test photos corresponding to the hemolysis rate (**Fig. 7c**), showing that Cu/PMCS had excellent biocompatibility.

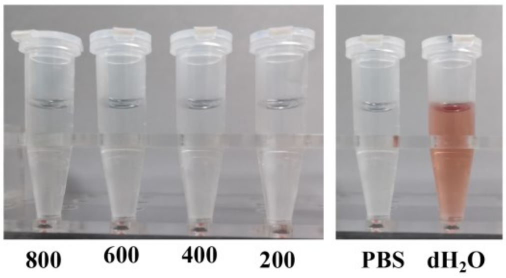

Fig. S35. Hemolysis test photos.

The results of blood tests indicated that Cu/PMCS had good biocompatibility *in vivo* (**Fig. S36**).

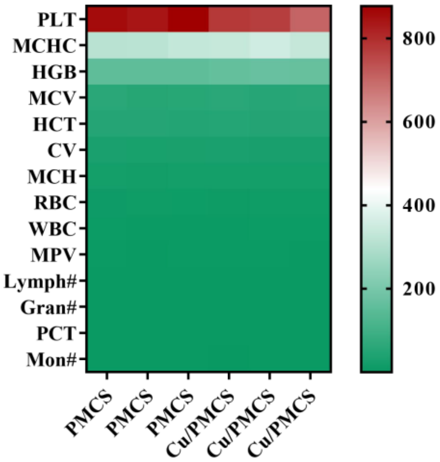

Fig. S36. Blood Test. Data represent means ± SD (n = 3).

There were no signs of toxicity in the histological analyses of major organ sections, including the heart, liver, spleen, lung, and kidney in H&E staining pictures (**Fig. S37**), indicating that Cu/PMCS had good *in vivo* biocompatibility.

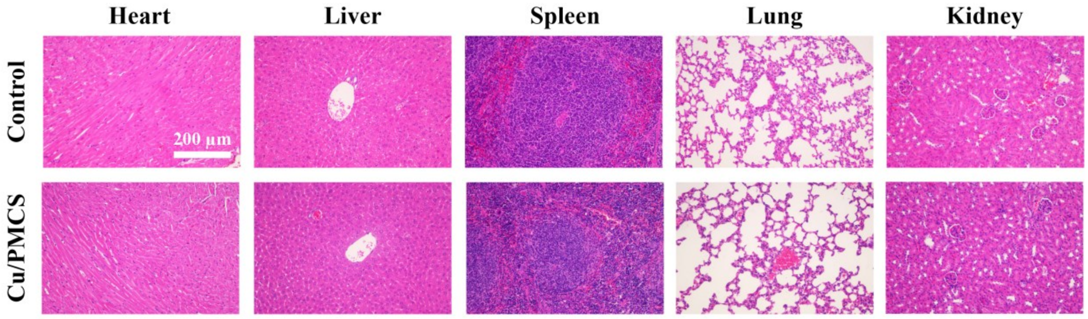

Fig. S37. H&E staining pictures of major organs in the mice.

As shown in **Fig. R38a**, Cu/PMCS promoted the migration of L929. Also, Cu/PMCS promoted the migrationof HUVECs (**Fig. R38b**) and accelerated angiogenesis of HUVECs (**Fig. R38c**). The quantitative results are consistent with qualitative results (**Fig. 7**), showing that Cu/PMCS has the potential to accelerate the wound healing *in vivo*.

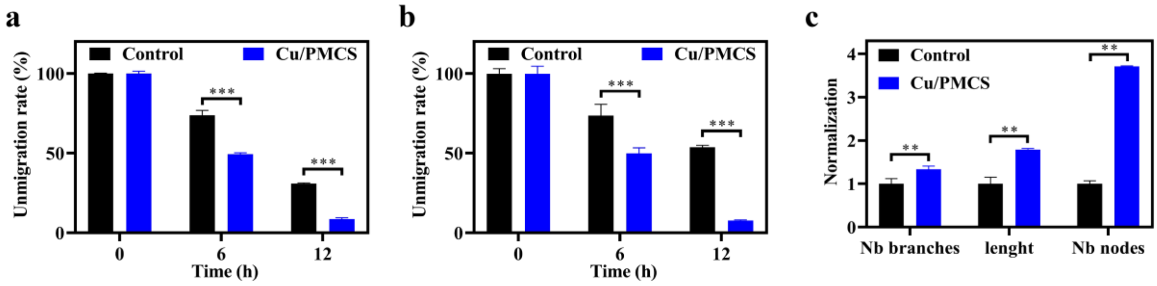

Fig. S38. Quantitative analysis of Cell migration in L929 (a), Cell migration in HUVECs (b), angiogenesis in HUVECs (c). Data represent means ± SD (n = 3). Statistical significance was calculated by two-way ANOVA analysis. $*p < 0.05$; $**p < 0.01$; $***p < 0.001$.

Fig. S39 is an infrared thermal image corresponding to the heating curve of mouse in wound model (**Fig. 9f**).

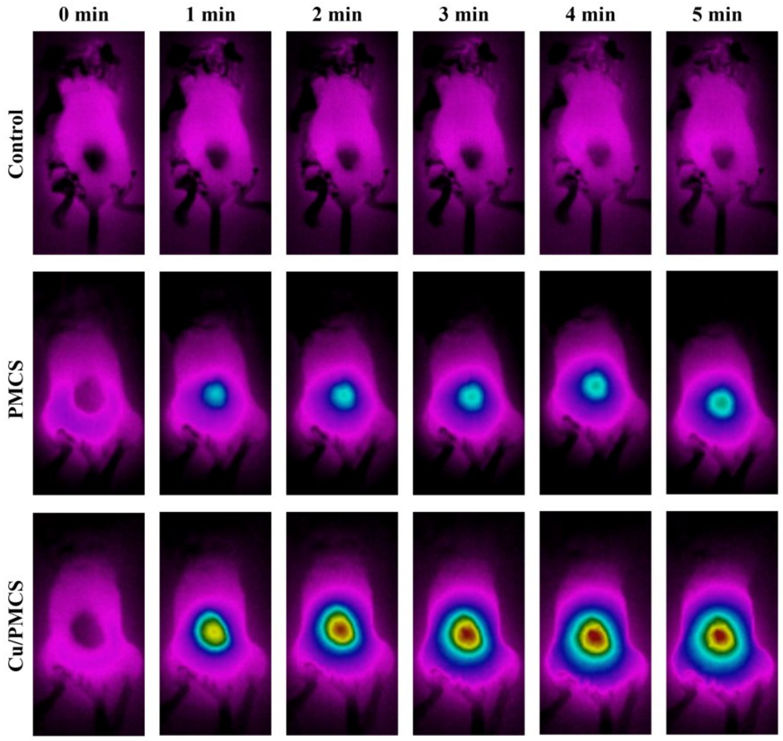

Fig. S39. Infrared thermographic images in wound model.

As shown in **Fig. S40a**, Cu/PMCS and Cu/PMCS+NIR group indicated the lower expression of CD68, which comparable to the qualitative results, showing that Cu/PMCS and Cu/PMCS+NIR inhibited the inflammatory response at the wound site effectively. The red signal in CD31 staining represented the expression of CD31, which is an endothelial cell marker used to assess vascular regeneration in the wound area. Compare with the Control and PMCS group, the expression of CD31 in Cu/PMCS and Cu/PMCS+NIR group is significantly increased and CD31 was most expressed in Cu/PMCS+NIR group (Fig. S40b). The above results are in line with qualitative results (**Fig. 7k**).

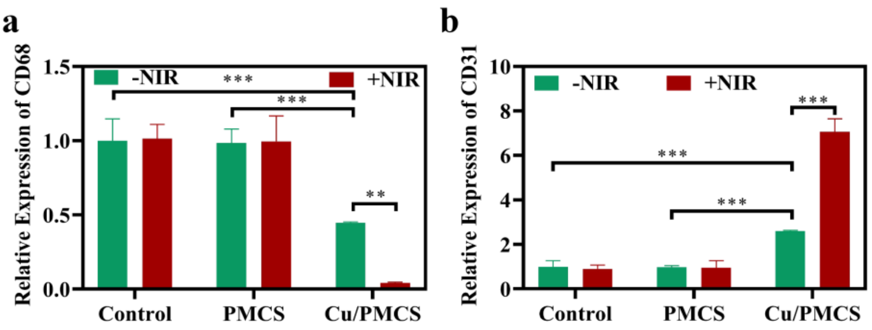

Fig. S40. Quantitative analysis of CD68 staining (a), and CD31 staining (b). Data represent means ± SD (n = 3). Statistical significance was calculated by two-way ANOVA analysis. *$p < 0.05$; **$p < 0.01$; ***$p < 0.001$.

3. Supplementary Tables

Table S1. EXAFS fitting parameters at the M K-edge for various samples.

Sample	Shell	CN^a	R(Å)^b	σ^2(Å^2)^c	ΔE_0(eV)^d	R factor
Zn K-edge (S_0^2=0.804)						
Zn foil	Zn-Zn	12*	2.64±0.01	0.0213±0.0091	-4.1±2.3	0.0151
ZnO	Zn-O	4.0±0.7	1.97±0.01	0.0037±0.0014	4.1±2.3	0.0131
	Zn-Zn	12.2±0.8	3.23±0.01	0.0250±0.0043	0.7±1.1	
Zn1	Zn-N	4.3±0.4	2.02±0.01	0.0087±0.0021	2.8±0.9	0.0075
Zn2	Zn-N/O	6.2±0.5	2.03±0.01	0.0104±0.0014	-1.1±1.0	0.0076
	Zn-Cu	1.2±0.2	3.13±0.01	0.0063±0.0035		
Cu K-edge (S_0^2=0.909)						
Cu foil	Cu-Cu	12*	2.54±0.01	0.0086±0.0004	4.5±0.5	0.0035
Cu2O	Cu-O	2.1±0.2	1.85±0.01	0.0034±0.0009	8.2±0.6	0.0200
	Cu-Cu	12.5±3.9	3.02±0.01	0.0201±0.0029		
	Cu-O	4.2±2.9	3.55±0.01	0.0091±0.0070		
CuO	Cu-O	4.2±0.2	1.95±0.01	0.0036±0.0005	7.8±0.9	0.0034
	Cu-Cu	5.0±0.5	2.94±0.01	0.0056±0.0046	-1.4±2.4	
	Cu-Cu	4.7±0.1	3.41±0.03	0.0056±0.0046		
Cu	Cu-N/O	5.6±0.6	1.95±0.01	0.0087±0.0019	8.4±1.5	0.0073
	Cu-Zn	1.3±0.2	2.90±0.02	0.0069±0.0047	-9.0±9.1	

^{a}*CN*, coordination number; ^{b}*R*, distance between absorber and backscatter atoms; $^c\sigma^2$, Debye-Waller factor to account for both thermal and structural disorders; $^d\Delta E_0$, inner potential correction; *R* factor indicates the goodness of the fit. $S0^2$ was fixed to 0.804 and 0.909, according to the experimental EXAFS fit of Zn foil and Cu foil by fixing CN as the known crystallographic value. Fitting range: $3.0 \le k$ (/Å) ≤ 12.5 and $1.0 \le R$ (Å) ≤ 3.0 (Zn foil and Cu foil); $3.0 \le k$ (/Å) ≤ 12.5 and $1.0 \le R$ (Å) ≤ 3.5 (ZnO); $2.0 \le k$ (/Å) ≤ 10.6 and $1.1 \le R$ (Å) ≤ 3.5 (Zn1); $3.0 \le k$ (/Å) ≤ 11.0 and $1.0 \le R$ (Å) ≤ 3.5 (Zn2); $3.0 \le k$ (/Å) ≤ 12.9 and $1.0 \le R$ (Å) ≤ 3.8 (Cu2O); $3.0 \le k$ (/Å) ≤ 9.0 and $1.0 \le R$ (Å) ≤ 3.5 (Cu). A reasonable range of EXAFS fitting parameters: $0.700 < S_0^2 < 1.000$; $CN > 0$; $\sigma^2 > 0$ Å^2; $\Delta E_0 < 10$ eV; R factor < 0.02.

Table S2. Atomic% of the different materials by XPS.

Atomic (%)	Zn	Cu	O	N	C
PMCS	1.37	0	5.85	11.79	80.91
Cu/PMCS-1	0.98	0.09	5.33	11.68	81.92
Cu/PMCS-2	0.98	0.16	5.48	9.88	83.50
Cu/PMCS-3	0.60	0.30	4.26	11.76	83.08

Table S3. ICP quantitative results.

	Zn^{2+} (wt%)	Cu^{2+} (wt%)
PMCS	6.23	0
Cu/PMCS-1	6.08	0.22
Cu/PMCS-2	4.06	0.6
Cu/PMCS-3	3.09	1.4

Table S4. Percentage of different types of N in XPS N1s.

	pyridimic N (%)	pyrrolic N (%)	graphitic N (%)	oxidized N (%)
PMCS	53.94	21.98	19.03	5.15
Cu/PMCS-1	55.58	19.78	19.38	5.26
Cu/PMCS-2	55.69	20.97	19.43	3.91
Cu/PMCS-3	55.23	15.18	19.38	10.21

Table S5. The release of Cu ions in Cu/PMCS at different time points

Time	Total content (ng/mL)	The release of Cu ions (ng/mL)	Percentage (%)
24 h	600	80.45	13.41
48 h	600	82.86	13.81

Table S6. Metal ratios for different sample preparations.

	$Zn(NO_3)_2{\cdot}6H_2O$ (mg)	$Cu(NO_3)_2{\cdot}3H_2O$ (mg)	Cu^{2+}: (Cu^{2+} + Zn^{2+})
ZIF	8940	0	0%
Cu/ZIF-1	8720	182	2.5%
Cu/ZIF-2	8490	363	5%
Cu/ZIF-3	8040	726	20%

Table S7. Primers sequences used in RT-qPCR analysis for angiogenic gene expression of L929.

Primer names	Forward sequence	Reverse sequence
VEGFA	GATCAGACCATTGAAACCAC	GAAGATGAGGAAGGGTAAGC
TGFB1	AACCAAGGAGACGGAATACA	CGTGGAGTTTGTTATCTTTGC
Col1a1	TGACTGGAAGAGCGGAGAGTA	GACGGCTGAGTAGGGAACAC
FGF2	TGACTGGAAGAGCGGAGAGTA	GACGGCTGAGTAGGGAACAC

Table S8. Primers sequences used in RT-qPCR analysis for angiogenic gene expression of HUVECs

Primer names	Forward sequence	Reverse sequence
VEGF	AGGGCAGAATCATCACGAAGT	AGGGTCTCGATTGGATGGCA

References

[1] J. Hafner, Ab-initiosimulations of materials using VASP: Density-functional theory and beyond, Journal of Computational Chemistry 29(13) (2008) 2044-2078.

[2] J.P. Perdew, K. Burke, M. Ernzerhof, Generalized Gradient Approximation Made Simple, Physical review letters 77(18) (1996) 3865-3868.

[3] G. Kresse, D. Joubert, From ultrasoft pseudopotentials to the projector augmented-wave method, Physical Review B 59(3) (1999) 1758-1775.

[4] A.D. Becke, Density- functional thermochemistry. IV. A new dynamical correlation functional and implications for exact- exchange mixing, J Chem Phys 104(3) (1996) 1040-1046.

[5] S. Grimme, J. Antony, S. Ehrlich, H. Krieg, A consistent and accurate ab initio parametrization of density functional dispersion correction (DFT-D) for the 94 elements H-Pu, J Chem Phys 132(15) (2010) 154104.

[6] P.E. Blöchl, Projector augmented-wave method, Physical Review B 50(24) (1994) 17953-17979.

[7] P. Yang, Y. Tian, Y. Men, R. Guo, H. Peng, Q. Jiang, W. Yang, Metal–Organic Frameworks-Derived Carbon Nanoparticles for Photoacoustic Imaging-Guided Photothermal/Photodynamic Combined Therapy, ACS Applied Materials & Interfaces 10(49) (2018) 42039-42049.

[8] M.-J. Dong, W. Li, Q. Xiang, Y. Tan, X. Xing, C. Wu, H. Dong, X. Zhang, Engineering Metal–Organic Framework Hybrid AIEgens with Tumor-Activated Accumulation and Emission for the Image-Guided GSH Depletion ROS Therapy, ACS Applied Materials & Interfaces 14(26) (2022) 29599-29612.
